# Modelling the memory of unmyelinated axons: Integration of a data‐driven approach with physiological memory concept

**DOI:** 10.1113/JP290729

**Published:** 2026-07-29

**Authors:** Anna Maxion, Jenny Tigerholm, Barbara Namer, Ekaterina Kutafina

**Affiliations:** ^1^ Joint Research Center for Computational Biomedicine Medical Faculty RWTH Aachen University Aachen Germany; ^2^ Scientific Center for Neuropathic Pain Aachen (SCN Aachen), Medical Faculty RWTH Aachen University Aachen Germany; ^3^ Center for interdisciplinary pain medicine, Department of Anesthesiology, Intensive Care, Emergency and Pain Medicine University Hospital Würzburg, Center for Interdisciplinary Pain Medicine Würzburg Germany; ^4^ Institute for Biomedical Informatics, Faculty of Medicine and University Hospital Cologne University of Cologne Cologne Germany

**Keywords:** activity‐dependent slowing, computational model, fibre memory, signal processing, unmyelinated axons

## Abstract

**Abstract:**

This study aims to present a simplified and resource‐efficient computational model for predicting activity‐dependent conduction velocity changes in unmyelinated axons, serving as a complementary tool to Hodgkin‐Huxley models. Our approach is based on the concept of ‘memory’, where the speed of action potentials is modulated by prior activity. We utilized microneurography data from 95 mechano‐insensitive C‐fibres of healthy human participants, including both sexes, across various stimulation protocols to optimize model parameters. The model incorporates linear long‐term and non‐linear short‐term memory components, effectively predicting propagation speed by convolving the history of recorded action potentials with the memory function. The proposed one‐dimensional and two‐dimensional memory functions yielded low mean squared errors in predicting the propagation speed of subsequent action potentials. This computational framework provides insights into dynamics of unmyelinated axons under varying conditions, enhancing our understanding of signal processing along the axon and its short‐term memory capabilities. Additionally our model demonstrates rapid computation times suitable for real‐time applications in electrophysiological experiments. This study introduces a novel model that simulates activity‐dependent conduction velocity changes in unmyelinated axons, which is crucial for effective signal processing during conduction. Unlike Hodgkin‐Huxley models that are computationally intensive and complex, our approach leverages fibre ‘memory’ to capture how prior activity influences conduction. With fewer parameters required to fit diverse datasets, including patient data, our highly efficient model enables faster simulations than Hodgkin‐Huxley models and facilitates the analysis of spike train propagation over long distances, and it is therefore suitable for modelling peripheral axons that extend up to 1 m.

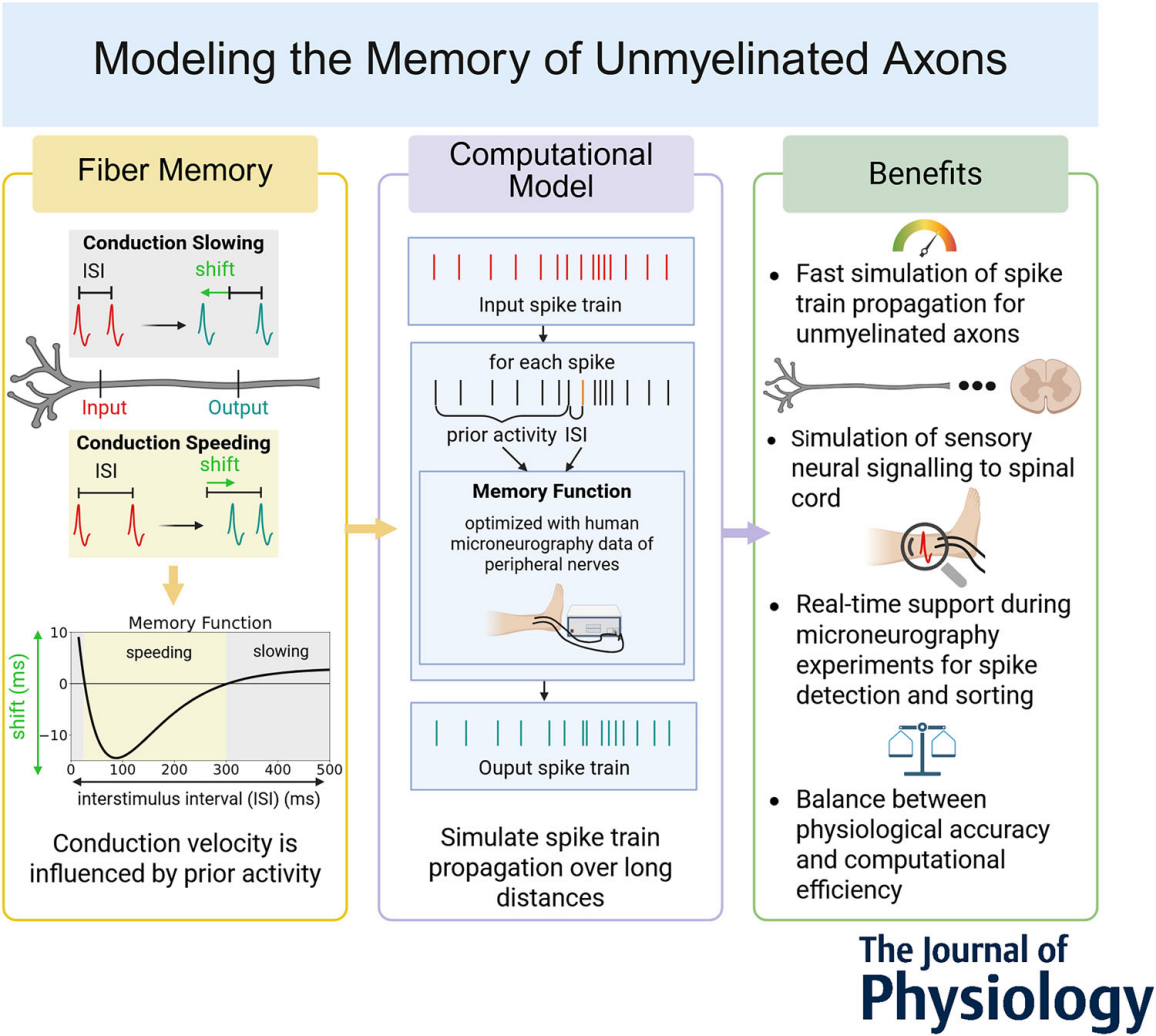

**Key points:**

- Unmyelinated axons, which are present in the peripheral and central nervous system, exhibit conduction velocity changes influenced by previous fibre activity, creating a form of fibre ‘memory’.
- This study presents a novel computational model that predicts conduction velocity changes in unmyelinated axons based on prior activity, providing a faster and more efficient addition to complex Hodgkin‐Huxley models.
- The new model incorporates both linear long‐term and non‐linear short‐term memory components, demonstrating rapid computation times suitable for real‐time applications.
- The model effectively captures the dynamics of nerve fibres, enhancing our understanding of axonal signal processing.
- This work offers insights into how previous activity influences axonal behaviour, informing future research on neurological disorders associated with altered nerve function.

## Graphical abstract

**Abstract figure legend** Simplified and resource‐efficient computational model for predicting activity‐dependent conduction velocity changes in individual spikes within spike trains in unmyelinated axons. **Fibre memory**: The conduction speed of each action potential is modulated non‐linearly by the axon's prior activity. A memory function can capture this dependence. **Computational model**: Spike trains serve as input into the model. For each spike the prior activity and the interstimulus interval to the preceding spike are supplied to the memory function, which is then convolved with the spike train to generate the propagated spike times along the axon. The memory function is optimized using microneurography data. **Benefits**: The model efficiently predicts spike timing over long axonal distances and enables real‐time applications due to its light‐weight architecture.

## Introduction

Understanding the function and dynamics of nerve fibres is fundamental to advancing our knowledge of the nervous system and developing treatments for neurological conditions. Unmyelinated axons play a crucial role in transmitting sensory signals, particularly in nociception and pain signalling (Dubin & Patapoutian, 2010). They are also abundantly present in the central nervous system (Soleng et al., 2003; Westenbroek et al., 1989).

An important aspect of the physiology of unmyelinated axons is characterized by activity‐dependent conduction velocity changes, such as activity‐dependent slowing (ADS) during repeated stimulation (Carr, 2013; Schmelz et al., 1995). ADS can be used to differentiate between different C‐fibre subtypes that exhibit characteristic conduction velocity changes in response to stimulation (Obreja et al., 2010; Serra et al., 1999; Weidner et al., 1999). The magnitude of ADS depends on several factors, including frequency, duration and intensity of previous activation of the axon (Ackerley & Watkins, 2018; Namer & Lampert, 2025; Weidner et al., 2000). When a fibre has already undergone substantial conduction velocity slowing, e.g. after prolonged activation with a high action potential (AP) count, additional stimulation can lead to a relative increase in conduction velocity rather than further slowing (Weidner et al., 2002), revealing complex adaptation dynamics depending on prior activity. Those adaptations lead to either increased or decreased frequencies arriving at the spinal cord and thereby provide a filter function of the axon, cutting out medium frequencies leading to a more bursting behaviour (Bucher & Goaillard, 2011).

Abnormal ADS patterns have been observed in inflammatory pain, neuropathic pain and small fibre neuropathies (Dickie et al., 2017; Kist et al., 2016; Kleggetveit et al., 2012; Namer et al., 2015; Ørstavik et al., 2003, 2006), making it a valuable marker for detecting and characterizing nerve dysfunction.

To study activity‐dependent conduction velocity changes microneurography is a valuable technique that allows direct recording of nerve fibre activity in awake humans (Ackerley & Watkins, 2018; Namer & Lampert, 2025; Schmelz & Schmidt, 2010; Vallbo, 2018). However microneurography is a tedious and time‐consuming procedure, requiring long experimental sessions to obtain sufficient data for analysis.

Computational models offer an alternative approach by simulating nerve fibre behaviour under different conditions. Complex multicompartmental models, including Hodgkin‐Huxley (HH) models, incorporate detailed biophysical dynamics of ionic currents and membrane potentials (Beeman, 2022; He et al., 2020; Petersson et al., 2014; Tigerholm et al., 2014). In our previous work (Maxion et al., 2023) we showed the capability of the HH approach to assess altered molecular mechanisms in patients. However this level of detail often results in long computation times, high computational resource demands and a complex parameter fitting process.

In our other work (Kutafina et al., 2022) we investigated a fully data‐driven approach to modelling of spike timing by relating the spiking history of the fibre to the magnitudes of activity‐dependent conduction velocity changes. This approach showed computational efficiency; however the physiological knowledge provided by the HH approach was missing.

Here we present a new model that balances the physiological sophistication of the HH model and the computational efficiency of a purely data‐driven approach by explicitly incorporating ‘memory’ of unmyelinated axons: the influence of past activity on current conduction latency using a convolution‐based approach inspired by geophysical wave propagation models (N. van Dalen et al., 2013). This is achieved by developing a ‘memory’ function that utilizes recovery cycle (RC) dynamics as its basis. These dynamics are governed by a refractory period, during which it is impossible or more difficult to generate an AP, followed by a supernormal phase where the next AP occurs faster, and then a subnormal phase where the subsequent AP is slower. The characteristics of these phases are influenced by previous activity (PA) and ADS. Furthermore these phases can be associated with potential underlying mechanisms: for instance the refractory period may relate to sodium channel inactivation, whereas the supernormal phase could be linked to the accumulation of extracellular potassium, and the subnormal phase might correspond to intracellular sodium accumulation. Therefore this framework allows for drawing mechanistic conclusions from the model and provides insights into the complex memory function of unmyelinated axons.

The proposed model is designed to be computationally fast and efficient by excluding detailed ion channel dynamics and other biophysical mechanisms. This makes it capable of simulating spike train propagation in longer axons and complex neural systems and could further make it a valuable tool to predict and sort spikes in real time during experiments. It simulates how the timing of APs changes while they propagate along the nerve fibre, which makes it suitable for predictive modelling and aiding in the analysis of experimental data. Therefore the model could enhance our understanding of nociceptive signalling mechanisms and contribute to a broader comprehension of central nervous system functions.

## Materials and methods

### Design of the computational model

**Figure 1:**
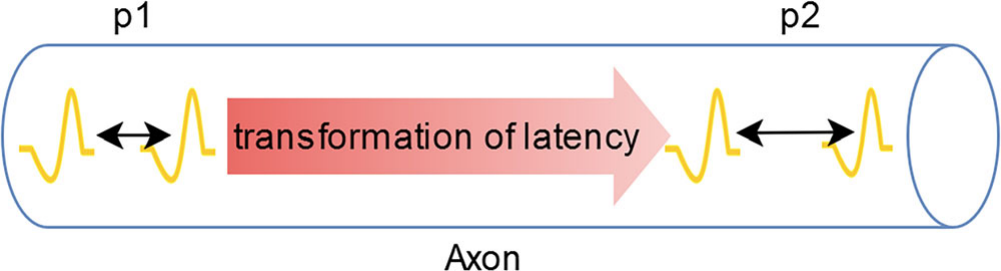
Underlying concept of the model The spike train generated at point p1 of the axon (e.g. in response to electrical stimuli) propagates along the axon to the measurement point p2. During this transmission the membrane properties are dynamically altered by preceding spikes. Consequently the travel times between p1 and p2 vary for each spike, leading to modulation of the temporal pattern of the original spike train.

We present a computational model developed to simulate axonal memory and predict changes in AP latencies in response to the spiking history. The model operates by taking an input spike train and applying a predefined memory function to adjust the latencies of subsequent spikes as they travel along the axon (Fig. 1). This transformation reflects the influence of prior activity on the timing of APs. The input spike train could also be interpreted as a stimulation signal, assuming that each stimulation pulse elicits exactly one AP and spontaneous activity is absent, which is characteristic behaviour observed in healthy C‐nociceptors.

The subsequent subsections detail the concept and modelling of axonal memory through RCs, the modelling of spiking history and the implementation of convolution techniques to capture the dynamic responses of unmyelinated axons. Further it is described what data were used to optimize and validate the model, as well as the optimization technique.

The computational model employs a mathematical function that represents axonal memory, which is convolved with a defined discrete signal that represents the history of previous spikes. This convolution allows for the prediction of relative latencies based on the activity history of the fibres. The model was implemented using Python, leveraging its computational capabilities to efficiently calculate the convolution and test various memory functions. This implementation provides a flexible framework for refining the model and adapting it to experimental data.

### Concept of axonal memory using recovery cycles

Unmyelinated axons exhibit activity‐dependent modulation of conduction velocity, which can be interpreted as a form of ‘memory’. This means that the conduction velocity at a given moment is influenced by the fibre's PA. The specific dynamic of this memory is dependent on the fibre type. This behaviour can be modelled using a memory function that reflects the fibre's recovery dynamics.

**Figure 2:**
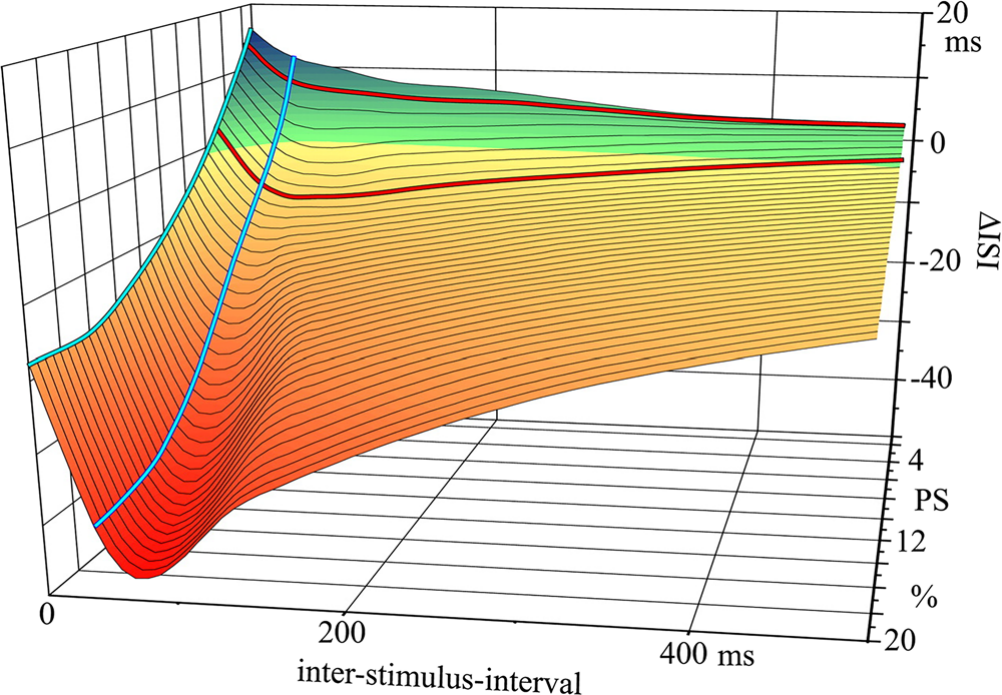
Presumed time course of postexcitatory effects in human C‐fibres This three‐dimensional figure summarizes the effects of pre‐existing slowing (PS, % decrease in conduction velocity) and interstimulus interval on ΔISI (supernormality in yellow‐red and subnormality in green). Supernormality of conduction is most pronounced at intense PS and short interstimulus intervals, whereas subnormality is observed at a low degree of PS. Blue lines represent findings from Weidner et al. (2002). Data for the red lines were taken from Weidner et al. (2000a). Figure and legend text were taken from Weidner et al. (2002). Copyright 2002 Society for Neuroscience.

A key example of this concept is provided by Weidner et al. (2002), which illustrates the presumed time course of postexcitatory effects in human C‐fibres (Fig. 2). The figure summarizes how pre‐existing conduction velocity slowing, expressed as the percentage decrease in conduction velocity, and the interstimulus interval following the last stimulation pulse influence the conduction velocity of the subsequent AP. The key observations include the occurrence of supernormality, characterized by the increased conduction velocity, at high pre‐existing slowing and short interstimulus intervals, whereas subnormality, or decreased conduction velocity, is observed at low pre‐existing slowing. For longer interstimulus intervals the effect on conduction velocity diminishes progressively, becoming negligible over time. Figure 10 depicts a similarly shaped model of memory function constructed for the purpose of our study.

Pre‐existing slowing serves as a measure of PA within the fibre: generally increased PA results in more conduction velocity slowing. Additionally the interstimulus interval (ISI) could also be interpreted as an interspike interval at the generation point of the spikes (Fig. 1, point p1), when we assume that each stimulus elicits exactly one spike and that there is no spontaneous activity.

To translate these findings into a functional model we defined a memory function that depends on ISI and/or PA. For long ISIs over 500 ms the effect decreases linearly, whereas for short ISIs under 500 ms supernormality occurs. This means the model axon has a non‐linear short‐term memory and a linear long‐term memory. In the two‐dimensional model the transition between sub‐ and supernormality is influenced by the PA value. This results in a piecewise function *m(t)* that captures both the activity‐dependent modulation of conduction velocity and its temporal recovery dynamics. The model is described in detail in the next sections.

### Modelling spiking history

To model the previous spiking activity we define a discrete signal function *s(t)* that assigns a value of 1 at specific points in time corresponding to the occurrence of spikes and 0 elsewhere. Mathematically this is expressed as:

$$s\left(t\right):=\delta \;\left(t\right)=\left\{\begin{array}{c} 1,\;t\;\in \;S\\ 0,\;else \end{array}\right.$$

where ***S*** represents the set of times at which spikes occur.

### Convolution model

To model the influence of previous spiking activity on the fibre's latency the signal *s(t)* is convolved with the memory function *m(t)*. The convolution operation is defined as:

$$c\left(t\right)=\left(m\times s\right)\left(t\right)=\int_{0}^{t}m\left(t-k\right)s\left(k\right)\;\mathrm{dk}\;,\;t\in S.$$

In this equation *m(t)* represents the fibre's memory dynamics, which encodes how PA affects the conduction latency at time *t* (see the Design of the computational model section for details). The convolution operation calculates the cumulative effect of all preceding activity on the latency.

At each time point *t* the function *m(t−k)* determines the weight of past events, meaning how strongly a spike occurring at time *k* contributes to the fibre's state at *t*. The signal *s(k)* indicates whether a spike occurred at time *k*, and its interaction with *m(t−k)* defines the effect of that spike on the fibre's conduction velocity. Integrating over all past time points combines the effects of all previous spikes, yielding the total memory‐driven modulation of conduction latency at *t*.

Importantly the conduction latency *c(t)* is only calculated for t∈S, i.e. at time points when spikes occur. This ensures that the latency is updated only in response to actual spiking events, rather than continuously evolving in time.

### Specific memory functions

The memory function is defined as a piecewise function that exhibits different behaviours depending on the length of the ISIs. For ISIs greater than or equal to 0.5 s we employed a linear memory function reflecting a linear decrease in conduction velocity for larger ISIs. In contrast for ISIs shorter than 0.5 s the function reflects supernormality, a phenomenon where conduction velocity temporarily increases following stimulation. The threshold of 0.5 s marks the point at which the supernormal phase in C‐fibres concludes across various conditions and background frequencies. Beyond this interval the fibre's response becomes linear (see Bostock et al., 2003). We implemented two specific memory functions: a one‐dimensional function that only depends on the ISI and a two‐dimensional function that depends on the ISI and the PA.

#### One‐dimensional memory function

The proposed memory function is only dependent on the ISI, meaning that the effect of PA on conduction latency is determined by the time elapsed since the last spike. The function is defined as:

(1) $$m\;\left(t\right)=\left\{\begin{array}{c} a-b\times t,\;t\geq 0.5\;s\\ exp\left(-d(4t-e\right))\;-c*\exp\left(-(4t-e\right)/f1))/120,\;t<0.5\;s \end{array}\right.\;$$

**Figure 3:**
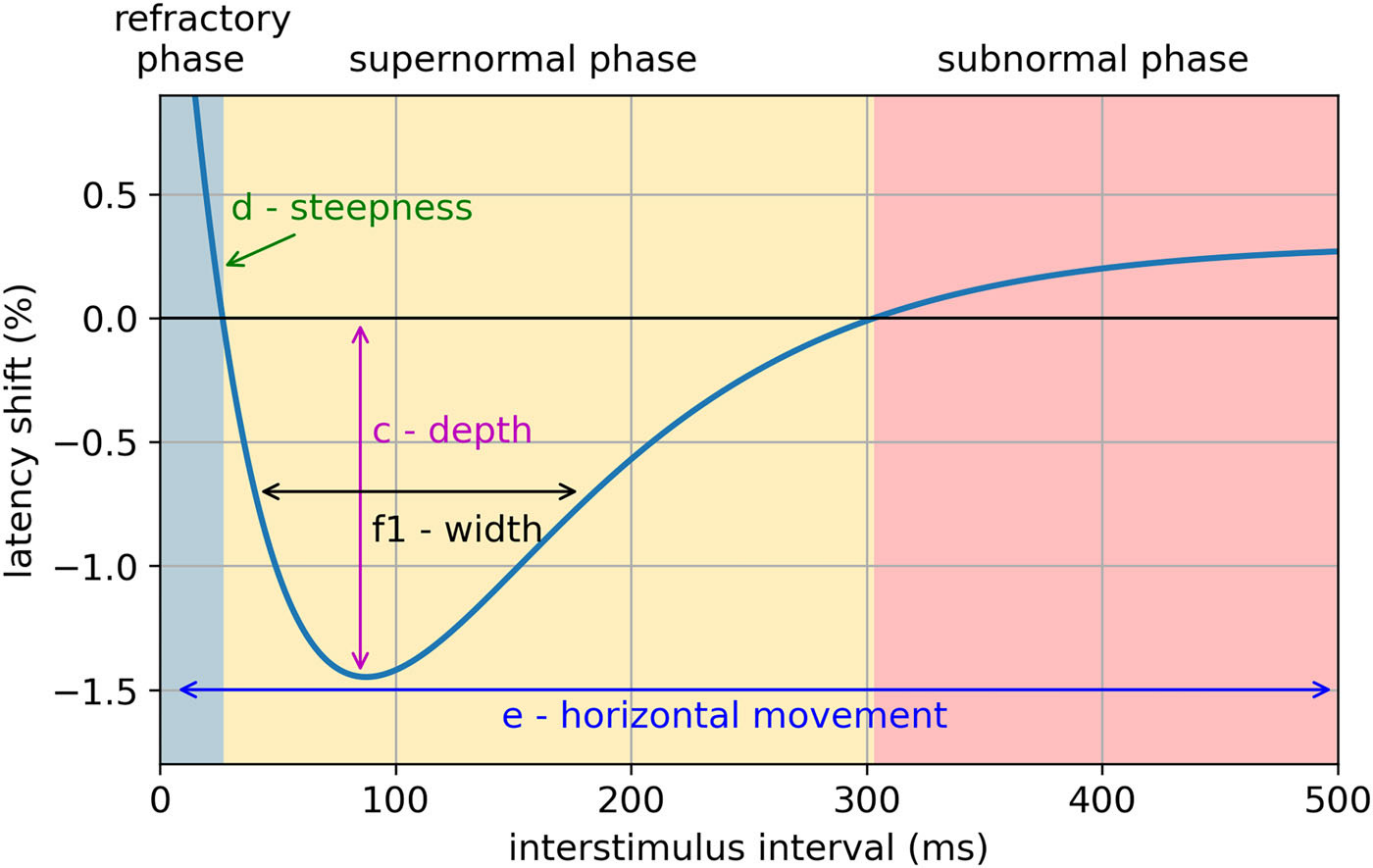
Visualization of parameters of the non‐linear part of the memory function and the corresponding phases The parameters explaining this function are highlighted with arrows in different colours. The parameter *c* (violet) controls the depth of the supernormal phase. The parameter *d* (green) controls the steepness of the beginning of the curve, corresponding to how fast the fibre recovers from the refractory phase. The parameter *e* (blue) moves the function horizontally, determining the position of the supernormal phase. Parameter *f1* controls the width of the supernormal phase.

This formulation describes how the fibre ‘remembers’ past activity, and how its conduction latency changes over time. The function shows three distinct phases that are also observed experimentally: a refractory phase, where it is difficult or impossible to generate another AP; a supernormal phase, in which the next AP travels faster than the preceding one; and a subnormal phase, reflecting a slowdown of subsequent spikes (Fig. 3).

For shorter ISIs (*t *< 0.5 s) the function is exponential, capturing the rapid and transient effects of recent stimulation. The term exp(−d(4t−e)) represents an overall exponential decline, and the term c∗exp(−(4t−e)/f1)) defines the supernormal phase. The contained parameters adjust the shape and duration of these phases as follows (Fig. 3): the parameter *e* moves the function horizontally, corresponding to a shift of the supernormal phase to different ISIs and reflecting the time course of all non‐linear processes. The parameter *d* determines the steepness of the beginning of the function, corresponding to how fast the fibre recovers from the refractory phase. The steepness of this slope can be interpreted as a measure of Na^+^‐channel inactivation, where a steeper slope suggests a faster recovery from inactivation. The parameter *f1* determines the width and *c* the depth of the supernormal phase, reflecting the extent of the ISIs with increased conduction velocity. The supernormal phase is physiologically linked to the accumulation of extracellular potassium. A pronounced supernormal phase, therefore, indicates a larger extracellular potassium accumulation.

For longer ISIs (*t *≥ 0.5 s) the memory function exhibits a subnormal phase, reflecting a slowdown of subsequent spikes and follows a linear decrease with increasing ISI, which can be adjusted with the parameters *a* and *b*. This describes that the influence of PA gradually fades over time, leading to a progressive normalization of conduction velocity, and can be associated with intracellular sodium accumulation.

#### Two‐dimensional memory function

Because the memory function is not only dependent on ISI (*t*) but also on the PA we add this complexity level to the model and propose the two‐dimensional modification of the memory function. It is defined as:

(2) $$m\;\left(t,\mathrm{PA}\right)=\left\{\begin{array}{c} a-b\times t+h\times PA,\;t\geq 0.5\;s\\ \left(\exp\left(-d\left(t-e_{1}\right)\right)\;-\log\left(q\times \mathrm{PA}\right)\;\times c*\exp\left(-f2\left(t-e_{2}\right)\right)\;\right)+g,\;t<0.5\;s \end{array}\right.\;$$

In the linear part of the function for larger ISIs (t≥0.5s) an additional term reflecting the dependence on PA has been incorporated as (h×PA).

For smaller ISIs (t<0.5s) the following modifications were made: the parameter *e* was divided into two distinct parameters $e_{1}$ and $e_{2}$, allowing independent movement of different parts of the function along the *x*‐axis. The parameter *h* was replaced with the term log(q×PA)×c, which makes the depth of the supernormal phase dependent on the PA. Thus larger values of PA result in a more pronounced supernormal phase. Although the logarithmic dependency was not explicitly stated in Weidner et al. (2002), it can be inferred from the plots presented in their study. Additionally we explored several alternative functions, including linear and square root functions. However the logarithmic function provided the best fit for our data.

The parameter *g* was introduced to allow for vertical adjustments of the entire memory function.

During the calculation of the convolution the PA is dependent on *t* and the set of stimulation pulses $S=\{s_{1},\;s_{2},\;\text{…},\;s_{n}\}$, n∈N and is defined as follows:

$$\mathrm{PA}=0,\;for\;t<s_{1}$$

$$\mathrm{PA}=c\left(s_{i}\right),\;\mathrm{for}\;s_{i}<t<s_{i+1},\;i>1$$

Before the first stimulation pulse is applied there is no pre‐existing activity. Therefore the fibre remains in its original state. Subsequently the PA is defined as prior latency. Therefore the memory function is technically only dependent on ISI (*t*), and the same convolution operation can be conducted as before.

### Overview of experimental data

#### Microneurography methodology

Microneurography is a technique to record nerve signals from individual C‐fibres. To achieve this an uninsulated microelectrode with a tip diameter of 0.005 mm (Microneurography needle, FHC, Bowdoinham, USA) is inserted into a fascicle of the superficial peroneal nerve at the ankle level, whereas a reference electrode is positioned superficially in the nearby skin. Electrical pulses, given by an isolated constant current stimulator (Digitimer DS7, Digitimer, Hertfordshire, UK), are applied to the receptive field of the fibres using superficially inserted electrodes (microneurography reference electrodes). This stimulation elicits APs in the terminal tree of the nerve fibres in the skin at a consistent latency, enabling the identification of multiple fibres within the same recording session. The mechanical thresholds of individual fibres are assessed using calibrated von Frey hairs and the marking method (Schmidt et al., 1995; Torebjörk & Hallin, 1974). Both the stimulation and the recording of fibre activity are performed using DAPSYS (Data Acquisition Processor System, Brian Turnquist, http://dapsys.net), which allows for real‐time data acquisition and processing.

#### Experimental microneurography data obtained in Namer's lab

Microneurography data from 97 mechano‐insensitive C‐fibres (CMi) of healthy individuals were used to optimize the computational model. Two fibres were excluded from this dataset: one due to incomplete data caused by recording issues and another because of misclassification in the original dataset. This resulted in a final dataset of 95 fibres.

The study adhered to the principles outlined in the Declaration of Helsinki and the statutory requirements of Germany. Ethical approval was obtained from the local ethics committees in Aachen (EK 141/19) and Erlangen (4361). All participants provided written informed consent to take part in the study.

For experimental stimulation each fibre was subjected to electrical rectangular pulses with a duration of 0.5 ms. Prior to each protocol electrical stimulation was paused for 2 min to ensure baseline stability.

**Figure 4:**
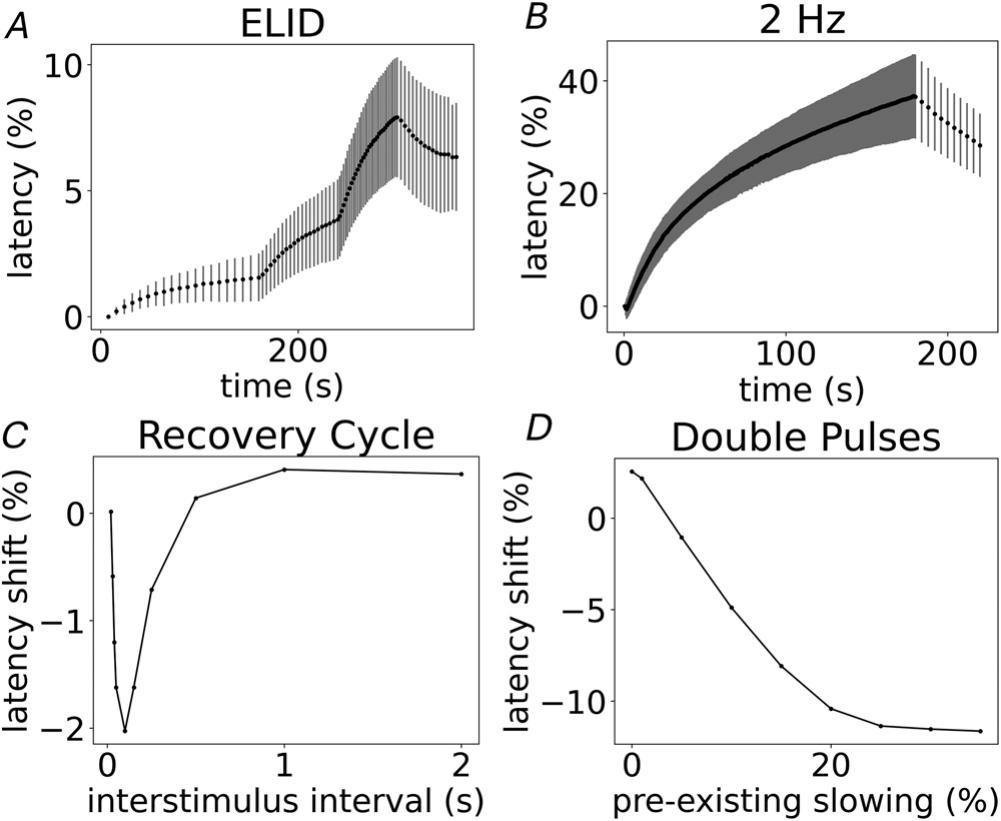
Overview of available data and stimulation protocols *A*, microneurography data from Namer's lab for electrical identification (ELID) protocol with mean and SD. *B*, microneurography data from Namer's lab for 2 Hz protocol with mean and SD. *C*, data from Weidner et al. (2000) for recovery cycle protocol. The latency shift is plotted for each interstimulus interval. *D*, data from Weidner et al. (2002) for double pulse protocol.

Two distinct stimulation protocols were employed:

1. The first protocol involved a series of rising pulse frequencies designed for fibre‐type discrimination, termed the electrical identification (ELID) protocol. This consisted of 20 pulses at 1/8 Hz, followed by 20 pulses at 1/4 Hz, then 30 pulses at 1/2 Hz and concluding with 10 pulses at 1/4 Hz (Fig. 4A).
2. In the second protocol each fibre was stimulated continuously at a frequency of 2 Hz for a duration of 3 min before being returned to a frequency of 1/4 Hz (Fig. 4*B*).

The microneurography data obtained from these protocols were subsequently analysed by extracting the latencies for all APs recorded during the ELID and 2 Hz stimulation. For each fibre the latencies were normalized to the base latency obtained after the initial 2 min pause. For further usage in the optimization process the mean latency across all fibres was computed.

#### Data from literature

In addition to our original dataset we incorporated findings from Weidner et al. (2000, 2002), which focuses on small interstimulus intervals. The data were extracted directly from figures in these papers using PlotDigitizer (https://plotdigitizer.com/).

Two distinct protocols were employed in these studies:

1. Recovery cycle: regular stimulation pulses at 1/4 Hz frequency were given until the latency stabilized. Subsequently additional pulses were interposed at varying intervals defined as follows: 2, 1, 0.5, 0.25, 0.15, 0.1, 0.05, 0.04, 0.03 and 0.02 s.
2. Double pulses: two stimulation pulses were delivered with an interval of either 20 or 50 ms, with each pair repeated at frequencies ranging from 1/16 to 1 Hz.

The RC data were obtained from Weidner et al. (2000), who examined 16 fibres. These data represent the mean latency shift of the base pulses in response to the extra stimulation pulses, plotted against the corresponding interstimulus interval (Fig. 4*C*).

The double pulse data were obtained from Weidner et al. (2002), Fig. 4, who examined a total of 51 fibres. These data represent the mean latency shift, i.e. the difference in latency between the second and first pulses of the pair, plotted against the PA, defined as the relative latency of the preceding AP (Fig. 4*D*).

### Parameter optimization

The optimization of the model function was conducted for the linear and non‐linear parts separately, each using stimulation protocols containing ISIs in the respective range.

The linear part of the memory function using ISIs > 0.5 s was optimized using the ELID and 2 Hz stimulation protocols. Both protocols exclusively contain frequencies within this range. The protocols were evaluated within the model, and the mean squared error (MSE) of the simulated latencies and the corresponding experimental data were minimized.

**Figure 5:**
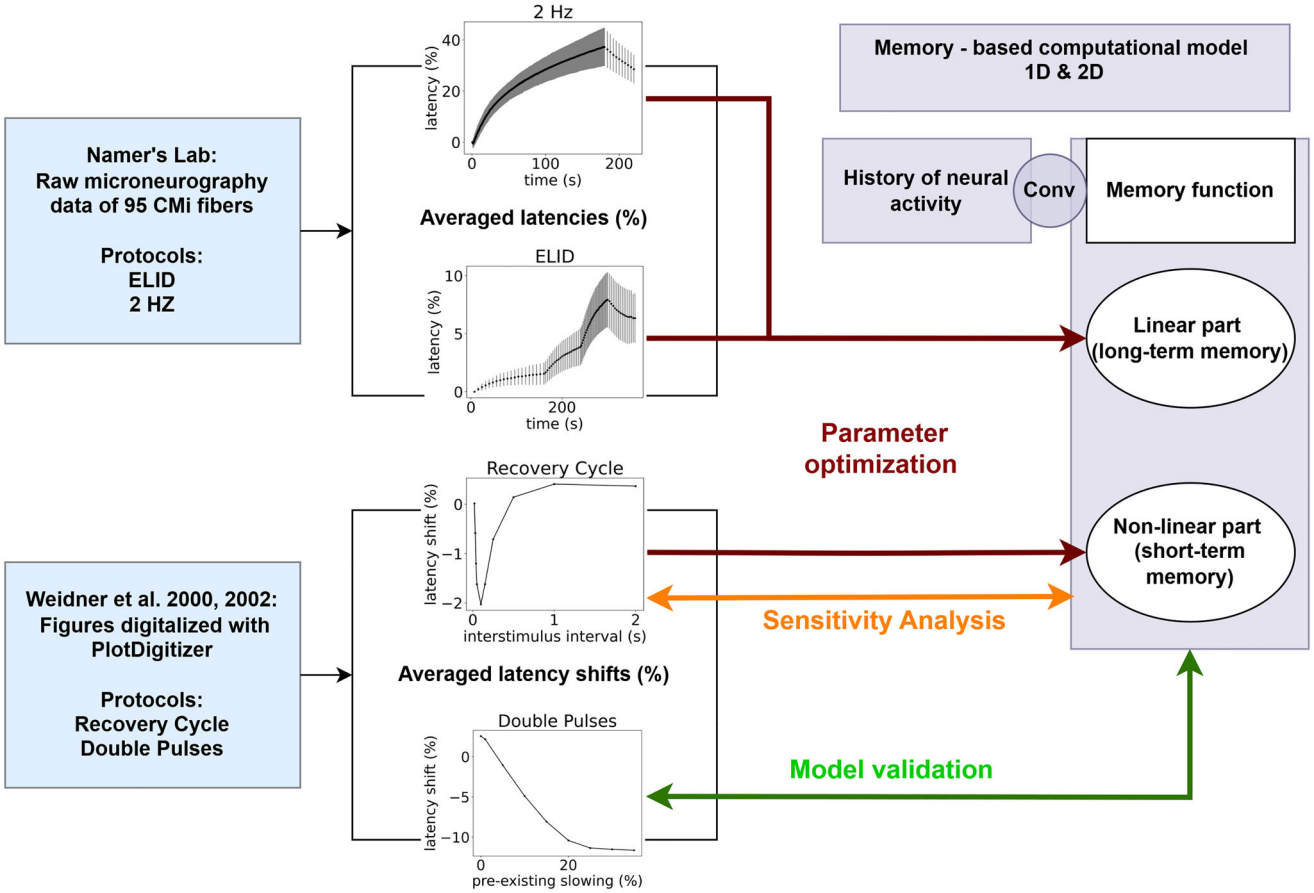
Overview of optimization process The linear part of the memory function, representing long‐term axonal memory, was optimized using microneurography data from Namer's lab that contained stimulation protocols with interstimulus intervals larger than 0.5 s. The non‐linear memory function, representing short‐term memory, was optimized using data from Weidner et al. (2000, 2002), with stimulation protocols that contained interstimulus intervals below 0.5 s. These data were further used for model validation and sensitivity analysis.

Once the parameters for the linear component were established the exponential part using ISIs ≤ 0.5 s was optimized. This phase utilized data from the RC protocol, comparing simulated latency shifts against experimental data by calculating MSEs across all shifts to assess model performance effectively. Figure 5 displays an overview of the optimization and validation process.

#### Optimization algorithm

For parameter optimization a particle swarm optimization (PSO) algorithm (Sedighizadeh et al., 2021) was used, which is a computational technique inspired by the social behaviour of organisms, such as swarms of birds or schools of fish.

The optimization process begins by randomly initializing the positions of 100 particles, where each particle represents a set of parameters for the memory function. These positions are constrained within predefined bounds, ranging from 0 to 50, depending on the parameter, which are chosen based on the expected range of parameter values. The selection of bounds ensures that the optimization process focuses on realistic solutions while allowing sufficient exploration of the parameter space.

For each particle the model's performance is evaluated by calculating a cost function, which quantifies the differences between the model's predicted latencies and the experimental latencies. The cost function is computed as the sum of the squared differences between these values. This approach provides a robust measure of how well the current parameter set aligns with the experimental data. The computation of all 100 particles was done in parallel with the high‐performance cluster of the RWTH Aachen.

The positions of the particles are updated iteratively using the generalized particle swarm optimization (GEPSO) algorithm (Sedighizadeh et al., 2021). This algorithm introduces enhancements to the standard PSO by incorporating additional factors into the position updates. Specifically each particle's position is adjusted based on the best position from a random particle, a random velocity component, the particle's current velocity, the particle's personal best position and the global best position of all particles in the swarm.

Compared to standard PSO GEPSO increases the diversity of particle movements and reduces the risk of premature convergence by getting stuck in a local minimum. These make it suitable to find solutions even in complex parameter spaces.

The optimization process continued for a fixed number of iterations, which was set to 1000 in this study. This stopping criterion balances computational efficiency with the need for sufficient iterations to achieve convergence.

To improve the efficiency of the optimization a two‐stage approach was employed. First the linear component of the memory function was optimized using larger frequencies of stimulation pulses. For this optimization the recorded microneurography data from the ELID protocol (frequencies ranging from 1/8 to 1/2 Hz) and the 2 Hz protocol were applied. Once the linear part was optimized the non‐linear component was refined using data from the RC protocol, which uses frequencies up to 66 Hz (interstimulus interval of 15 ms). Non‐linear effects only occur during shorter intervals, necessitating a distinct protocol to accurately capture these dynamics. Further this stepwise approach ensures that the simpler aspects of the model are fine‐tuned before addressing the more complex, non‐linear dynamics.

### Validation

The double pulse protocol and modified RC protocols with different background frequencies and interstimulus intervals were used to validate the model. For the double pulse protocol the MSE of the latency shifts was calculated to assess the agreement between the model's predictions and the experimental data. For the modified RC protocols no experimental data were available for direct comparison.

To evaluate the generalizability and predictive performance of the computational model we validated it using stimulation protocols that were not included during the optimization process. These protocols were selected to provide diverse input conditions and ensure the model's ability to predict responses beyond those it was directly trained on. The stimulation protocols used encompass a wide range of PAs and interstimulus intervals to capture the diverse conditions typically encountered in microneurography experiments.

### Additional protocols for sensitivity analysis

In addition to the primary protocols augmentary stimulation protocols were implemented as proof of concept for which no existing data for comparison are available. These additional protocols were based on the RC protocol. The first set of protocols varied the interstimulus intervals of the extra pulses. One protocol introduced noise to the ISIs, resulting in slight variations from the original intervals. The other protocol utilized ISIs that fell between the original values, specifically 1.5, 0.75, 0.375, 0.2, 0.125, 0.075, 0.045, 0.035 and 0.025 s. The second set of augmentary protocols explored varying frequencies for the background pulses, ranging from 1/20 to 1/2 Hz.

The implementation of augmentary stimulation protocols enhances the robustness and applicability of our computational model. By exploring variations in ISIs and background pulse frequencies we aim to assess the model's sensitivity to changes that may occur in real‐world scenarios. Given that existing data for comparison are lacking these additional protocols provide an opportunity to investigate the impact of different stimulation patterns on AP dynamics.

## Results

### One‐dimensional memory function

#### Parameter optimization

**Figure 6:**
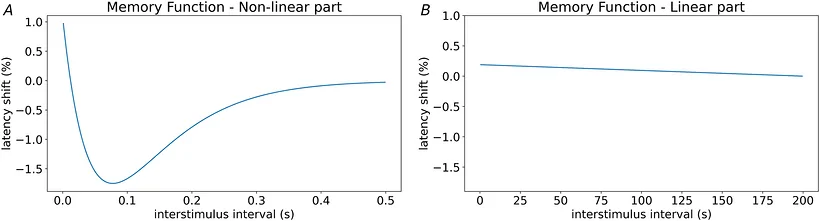
Illustration of the one‐dimensional memory function with recovery cycle dynamics *A*, the memory function for interstimulus intervals up to 0.5 s shows behaviour similar to recovery cycle dynamics with a supernormal phase. *B*, the memory function for interstimulus intervals larger than 0.5 s shows a linear behaviour.

The optimization of the one‐dimensional memory function was conducted using PSO in two steps. In the first step the linear component of the memory function was optimized. The obtained parameter values were *a* = 0.19 and *b* = −0.00095. In the second step the parameters for the exponential component of the function were refined. The PSO algorithm yielded values of *c* = 12, *d* = 4.69, *e* = 1.59 and *f*1 = 0.33. The final memory function is illustrated in Fig. 6.

#### Model performance

**Figure 7:**
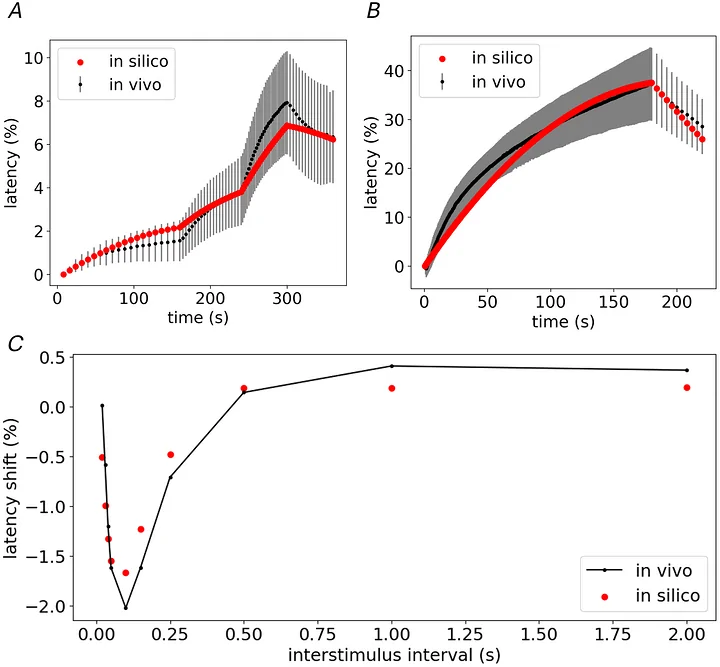
Model performance of the one‐dimensional memory function *A* and *B*, simulated latency increase expressed as a percentage (red) for the electrical identification (ELID) and 2 Hz stimulation protocols, respectively. These are compared to latencies obtained from our own microneurography data (black), which display the mean and SD. *C*, relative latency shifts, also expressed as a percentage, calculated from the simulated latencies of the computational model (red) for the recovery cycle protocol. These shifts are compared to data from Weidner et al. (2000) (black), showing the mean values.

The performance of the convolution model utilizing the one‐dimensional memory function was evaluated through its application to the ELID, 2 Hz and RC stimulation protocols. Figure 7 illustrates the simulated latencies compared to microneurography data from our own database (ELID and 2 Hz), as well as data from Weidner et al. (2000, 2002) (RC). The MSE was calculated for each case, yielding a value of 4.24 for 2 Hz stimulation and 0.41 for the ELID protocol (Table 1). For the RC protocol the comparison between Weidner's data and the simulated data resulted in an MSE of 0.09.

**Table 1:** Mean squared errors (MSEs) for the computational model using one‐dimensional and two‐dimensional memory functions across various stimulation protocols

	One dimensional	Two dimensional
ELID	0.41	0.41
2 Hz	4.24	3.00
Recovery cycle	0.09	0.027
Double pulses 20 ms	12.20	1.87
Double pulses 50 ms	51.14	17.45

*Note*: Note that MSE values are only comparable between the different models for each protocol and should not be compared across different protocols.

#### Validation

**Figure 8:**
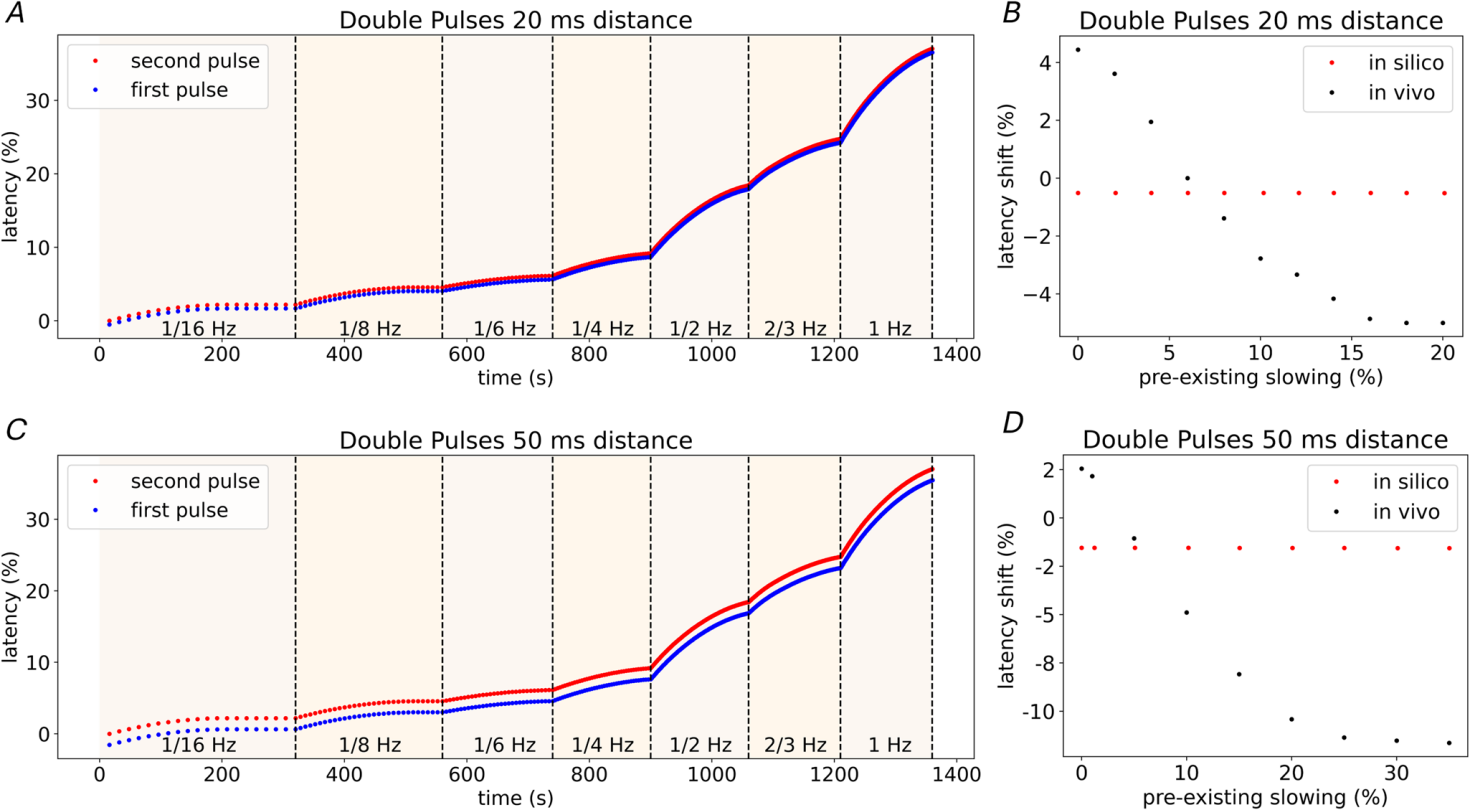
Results of the computational model for the double pulse protocols using the one‐dimensional memory function Two pulses are given at 20 or 50 ms distance at frequencies ranging from 1/16 to 1 Hz. *A* and *C*, relative latency over the time of the stimulation. *B* and *D*, The pre‐existing activity state, represented as the relative latency of the preceding action potential, is compared to the latency shift, defined as the difference between the preceding and the current action potential. The real data are taken from Weidner et al. (2002).

The model was validated using the double pulse protocol, which included interstimulus intervals of 20 and 50 ms at varying frequencies (Fig. 8). As the frequency of the pulse pairs increases, resulting in greater PA, the latency difference between the two pulses remains constant. However this behaviour deviates substantially from what would occur in a real fibre, where experimental data indicate that increasing PA leads to a decrease in the latency shift between the pulses, which highlights the limitations of the one‐dimensional model This discrepancy suggests that the current model lacks the ability to represent the full range of dynamic changes that occur with varying PA.

### Two‐dimensional memory function

#### Parameter optimization

**Figure 9:**
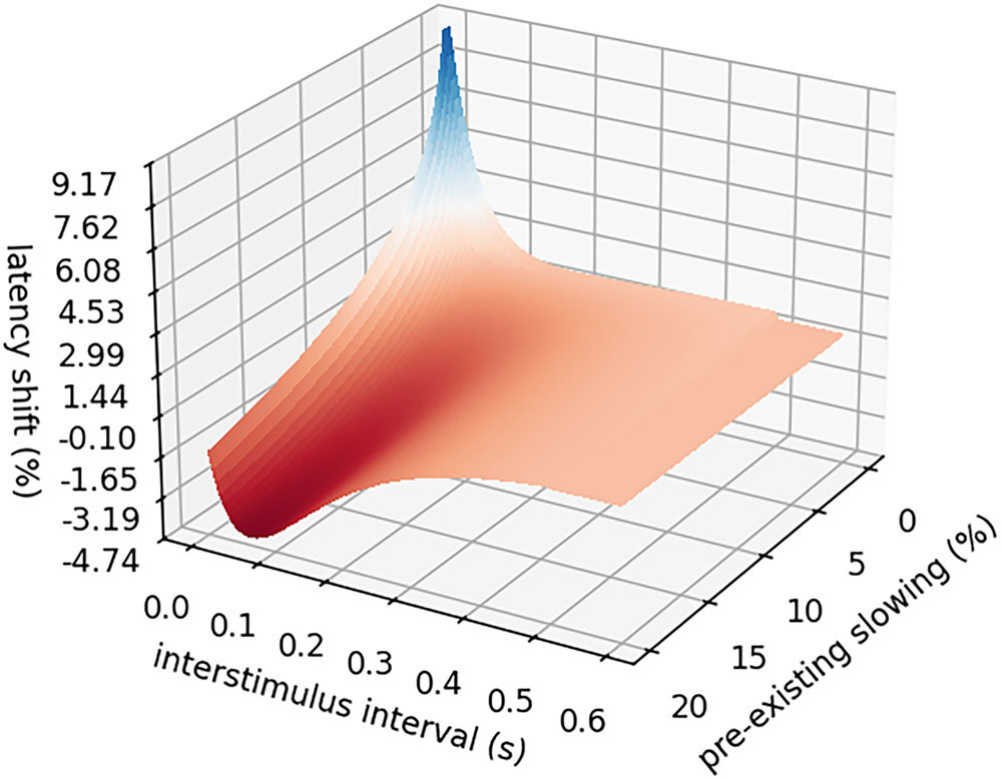
Illustration of the two‐dimensional exponential memory function The memory function predicts the latency shift (%) of an action potential given the interstimulus interval (s) and the pre‐existing activity state (%). Red coloring indicates a negative latency shift (i.e., speeding of the subsequent action potential), whereas blue coloring indicates a positive latency shift (i.e., slowing of the subsequent action potential). For interstimulus intervals below 0.5 s the function shows a supernormal phase for increased pre‐existing slowing. The function reproduces the dynamics shown in Figure 2 from Weidner et al. (2002).

The parameters of the two‐dimensional memory function (eqn (2)) were optimized using PSO, following the same methodology applied to the one‐dimensional function. This optimization was conducted in two steps: first we focused on optimizing the linear component, resulting in values of *a* = 0.22, *b* = 0.0014 and *h* = 0. In the second step we refined parameters for the non‐linear part of the function, yielding values of *d* = 21.800, *e*1 = 0.105, *e*2 = 0.0, *f*2 = 7.530, *g* = 0.553, *c* = 2.851, *q* = 3.485. The resulting memory function is illustrated in Fig. 9.

**Figure 10:**
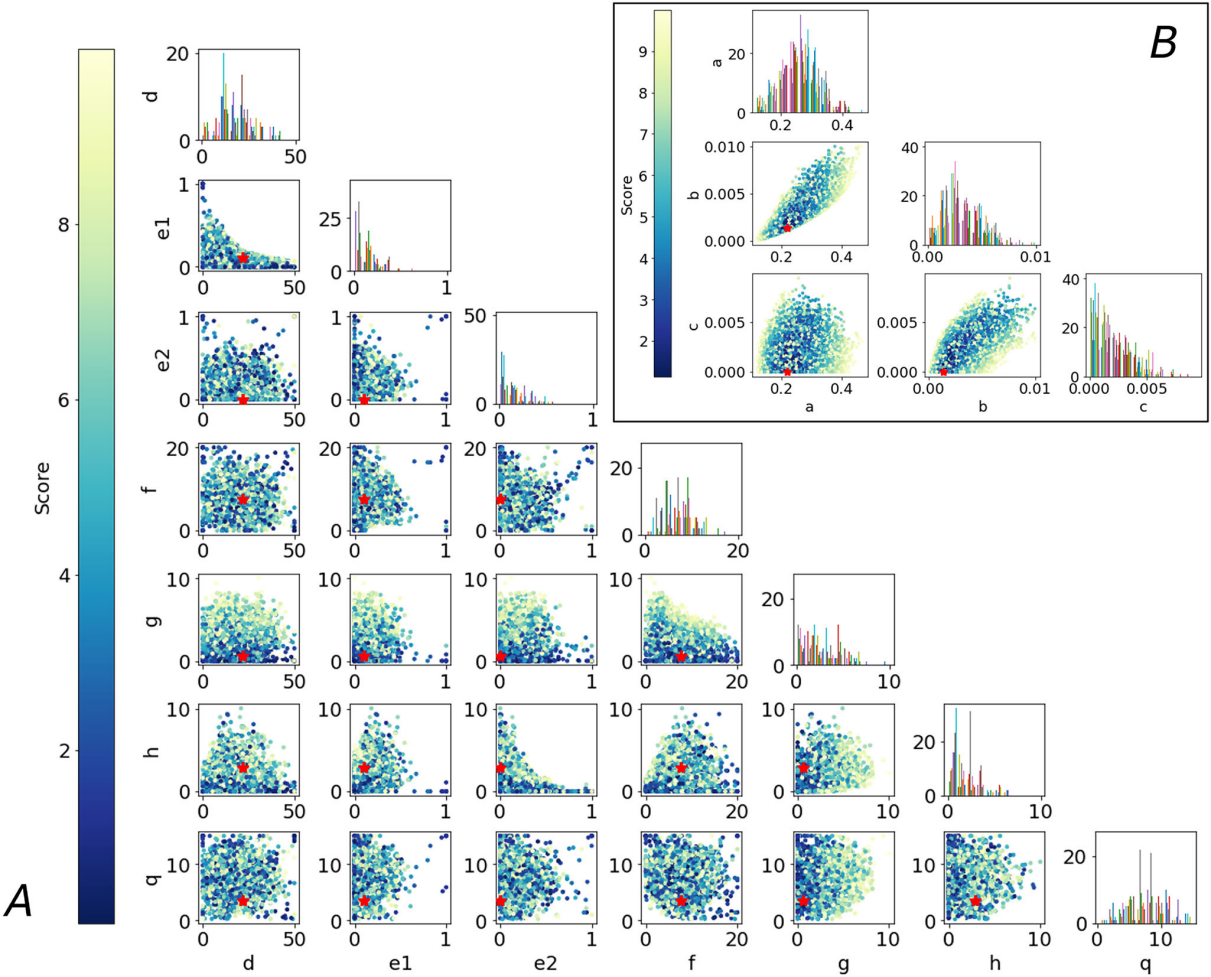
Results of the parameter optimization using a particle swarm algorithm Each dot represents one particle, i.e. one evaluation of the model for a specific parameter set. The colour indicates the fit/score of the model, with lower values indicating a better fit. Only particles with scores below 10 are shown. The red star marks the best particle, i.e. the particle with the lowest score. The histograms show how many particles were evaluated in each parameter region. *A*, parameter optimization for the non‐linear component of the memory function. *B*, parameter optimization for the linear component of the memory function.

In Fig. 10 the optimization process is illustrated, showing the scores of all evaluated particles. The distribution of evaluated particles revealed clusters of high‐performing parameter sets, suggesting specific regions of parameter space that contribute most effectively to model performance.

#### Model performance

**Figure 11:**
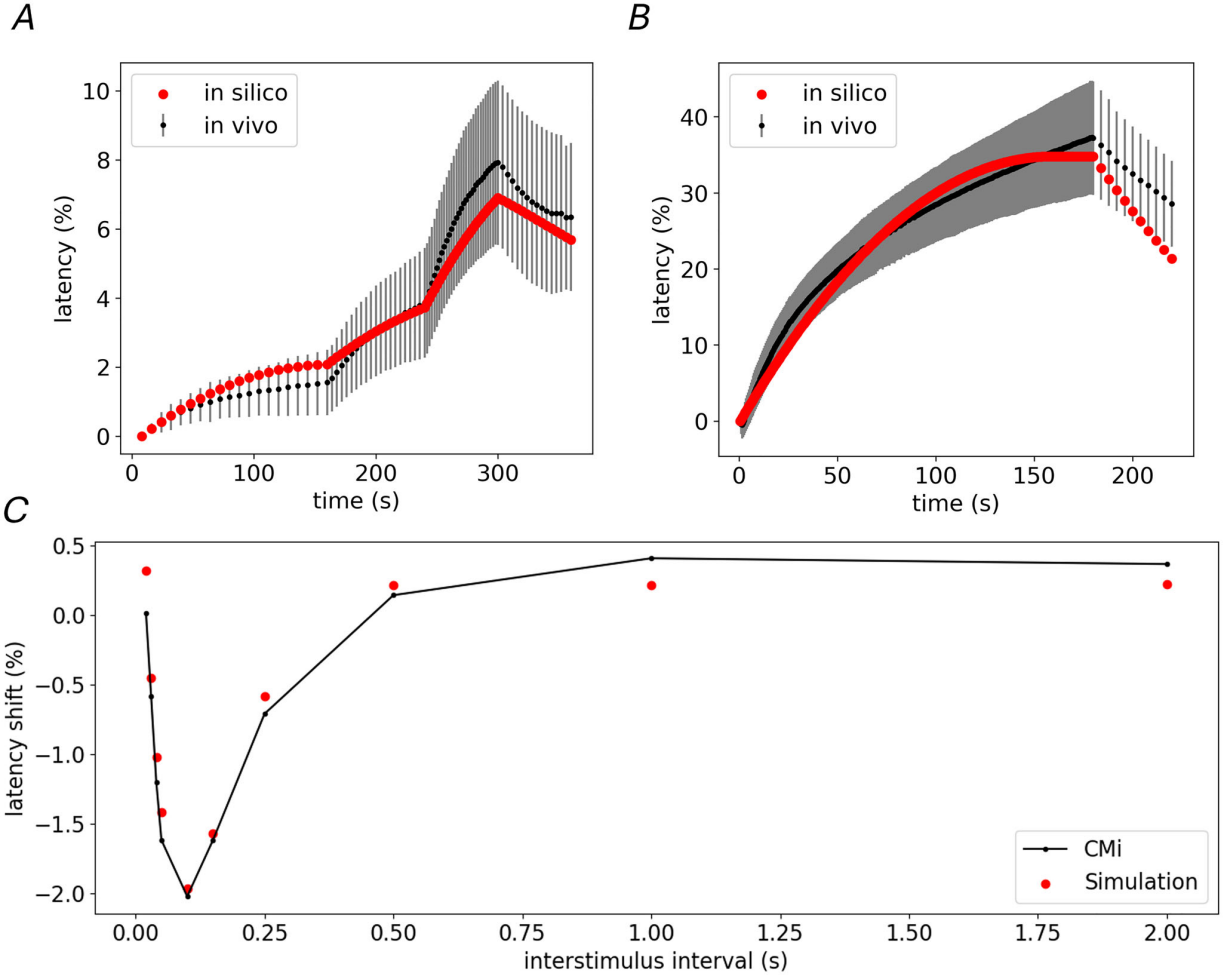
Model performance of the two‐dimensional memory function *A* and *B*, simulated latency increase expressed as a percentage (red) for the electrical identification (ELID) and 2 Hz stimulation protocols, respectively. These are compared to latencies obtained from our own microneurography data (black), which display the mean and SD. *C*, relative latency shifts, also expressed as a percentage, calculated from the simulated latencies of the computational model (red) for the recovery cycle protocol. These shifts are compared to data from Weidner et al. (2002) (black), showing the mean values.

To assess the performance of the model we evaluated its predictive accuracy across different protocols. The MSE for the ELID protocol is 0.41, whereas for the 2 Hz protocol it stands at 3.00 (Table 1). The memory function captures the behaviour of the postexcitatory effects well and preserves defining features, e.g. an exponential behaviour for low PA and the characteristic dip for low ISIs. The model is able to replicate the RC well with an MSE of 0.027 (Fig. 11).

#### Validation

**Figure 12:**
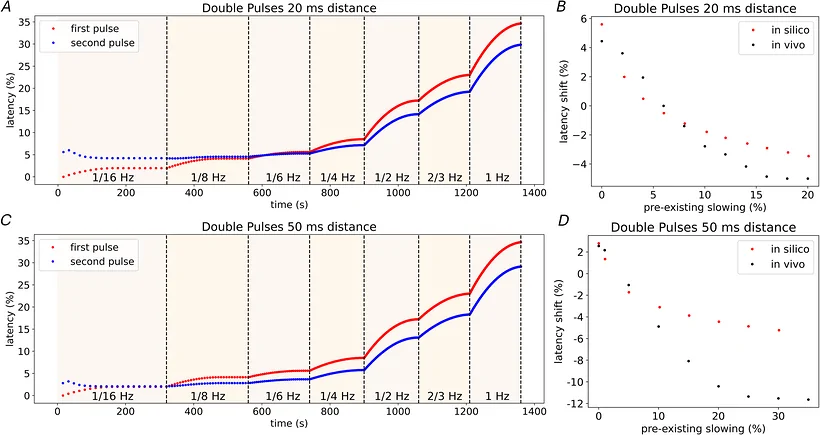
Results of the computational model for the double pulse protocols using the second two‐dimensional memory function. Two pulses are given at 20 or 50 ms distance at frequencies ranging from 1/16 to 1 Hz *A* and *B*, relative latency over the time of the stimulation. *B* and *D*, the pre‐existing activity state, represented as the relative latency of the preceding action potential, is compared to the latency shift, defined as the difference between the preceding and the current action potential. The *in vivo* data are from Weidner et al. (2002).

The last protocol used for validation is the double pulse protocol, where two pulses at 20 or 50 ms distance are given at different frequencies ranging from 1/16 to 1 Hz (Fig. 12). For higher frequencies the second pulse shows activity‐dependent speeding, whereas for lower frequencies the second pulse is relatively slower than the first pulse. The computational model was able to capture the dynamics well, even though the magnitude of the changes differs from the real data, especially for distances of 50 ms.

#### Sensitivity analysis

**Figure 13:**
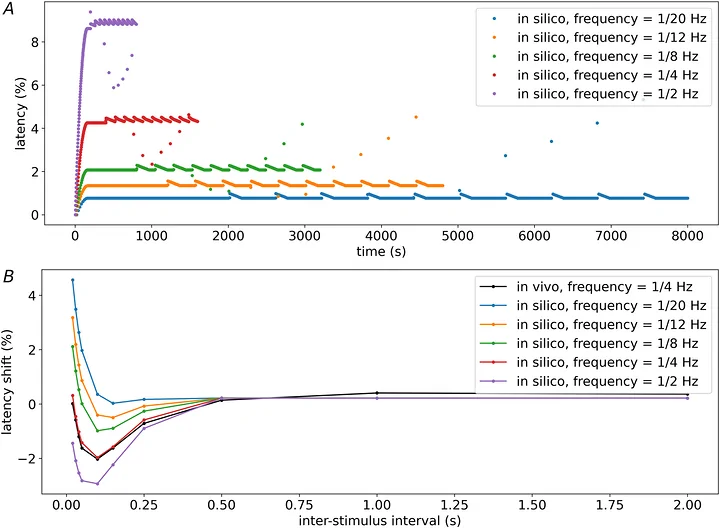
Sensitivity analysis of the two‐dimensional memory function The recovery cycle protocol was simulated with varying frequencies of the background pulses between 1/2 Hz and 1/20 Hz to achieve different levels of pre‐existing activity. *A*, latency of the action potentials in percent over the time of the stimulation in seconds. *B*, the interstimulus interval (s) is shown compared to the relative latency shift (%) induced by the extra pulses. In black, values of the real data from Weidner et al. (2002) are given for a background frequency of 1/4 Hz. The supernormal phase increases for higher frequencies of background pulses, i.e., higher pre‐existing activity.

Further the RC protocol was adapted with different background frequencies that induce a different level of PA (Fig. 13). The background frequencies ranged from 1/20 to 1/2 Hz. To account for variability in stabilization points across different frequencies we initiated each simulation with 100 pulses at 1/4 Hz before interposing additional pulses. A higher frequency of the background pulses induced a higher PA, which led to a more pronounced supernormal phase in the RC.

## Discussion

We developed an innovative computational model for axonal AP conduction based on the memory function of an axon. This model convolves a memory function with a signal function that contains the AP initiation times. It predicts the conduction velocity of unmyelinated axons efficiently and fast and thus is especially useful for time‐sensitive and intensive simulations, such as simulating complex neural networks, real‐time spike sorting during experiments or parallel assessments of synchronicity.

In this project we explored two potential memory functions: a one‐dimensional function that is dependent on the inter‐stimulus intervals and a two‐dimensional function that is additionally dependent on the PA. Both memory functions have in common a linear component for larger ISIs and a non‐linear component for shorter ISIs.

The model is validated using stimulation protocols that were not used for optimization and were chosen to cover a wide range of ISIs and a variety of PAs to cover the whole parameter space.

### One‐dimensional memory function

The one‐dimensional memory function only depends on the ISI and is defined as a piecewise function with different dynamics for ISIs below and above 0.5 s. When APs are elicited with an ISI greater than 0.5 s, only ADS of conduction velocity is observed. In contrast for ISIs below 0.5 s additional effects related to the RC can occur, leading to reduced ADS or even speeding of conduction velocity. The model shows a low MSE for the ELID and 2 Hz protocols (Table 1).

During the RC protocol the model could replicate the supernormal phase, which plays a critical role in the dynamics of nerve fibres and can be altered in patients (Namer et al., 2015). Additionally the model demonstrated robustness even when random variations were introduced to the existing RC protocol (see Figure S1 in the supplementary material), indicating its reliability under various conditions.

However the one‐dimensional memory function does not account for PA, leading to the same results regardless of the amount of pre‐existing activity, as observed in the results for the double pulse protocol (Fig. 8) and in a sensitivity analysis, which can be found in the supplementary material.

### Two‐dimensional memory function

To solve the limitations of the one‐dimensional memory function the two‐dimensional memory function includes the PA as an additional variable. Defined as a piecewise function it exhibits different dynamics for ISIs below and above 0.5 s, with a two‐dimensional linear function applied for larger ISIs. Parameter optimization revealed that the PA could be set to zero, indicating that for ISIs greater than 0.5 s our model can accurately predict latencies using only the ISI of stimulation pulses, without explicitly considering prior activities, as ISIs inherently contain information about PA.

The component of the memory function for small ISIs consists of two exponential components subtracted from each other. Additionally the second exponential term is multiplied by the logarithmic term and thus balances its impact. The resulting memory function closely resembles the function from Weidner et al. (2002) and can accurately reproduce a wide variety of stimulation protocols with different amounts of pre‐existing activity and covering a wide range of ISIs.

The MSE of the two‐dimensional model was smaller than or equal to that of the one‐dimensional model for all protocols (Table 1).

The ELID and 2 Hz protocols utilize stimulation frequencies at or below 2 Hz and thus evoke mainly ADS, correlating with the number of preceding APs. The main reason for ADS is thought to be intracellular sodium accumulation (Tigerholm et al., 2014), which constantly accumulates with each AP, thereby generating most likely a linear effect. Thus by modelling ADS with our approach we can also conclude on possible molecular processes underlying certain biological phenomena.

Stimulation protocols that include smaller ISIs, such as the RC protocol and the double pulse protocol, are reproduced utilizing additionally the non‐linear component of the function, indicating that non‐linear mechanisms play an important role in these fast dynamics. This could be due to a combination of different processes, e.g. sodium channel inactivation dependent on the membrane potential in combination with activity‐dependent hyperpolarization (Bostock et al., 2003; Bucher & Goaillard, 2011) and changes in intra‐axonal and extra‐axonal ionic concentrations (Tigerholm et al., 2015). The RC dynamics enhance the contrast of high and low activity in nerve fibres by modulating their timing, delaying them when prior activity is minimal and accelerating them when prior activity is substantial, resulting in a bursting pattern at the spinal cord (Bucher & Goaillard, 2011).

Further the model could capture the dynamics introduced by different amounts of pre‐existing activity observed in the double pulse protocol, where the overall dynamics were captured well even though the magnitude of the changes differed from the real data. In our model this dynamic is governed by the logarithmic increase in the supernormal phase depending on the pre‐existing activity. This indicates that a logarithmic function does not fully capture the fibre dynamics, and another type of dependency may be needed in future iterations of the model to more accurately represent these variations.

For increased background frequency in the RC protocol, which relates to more pre‐existing activity, the supernormal phase of the RC increases in accordance with the behaviour of real C‐fibres (Bostock et al., 2003). Introducing noise in the RC protocol showed that the model maintained its accuracy, highlighting its robustness in varying conditions (see Figure S2 in the supplementary material).

In conclusion the model demonstrates robust agreement with experimental data, and the optimized parameter set captures the essential physiological mechanisms underlying the observed behaviour of unmyelinated axons. It captures key physiological phenomena, including different reactions to different stimulation frequencies and different levels of PA, both of which are critical for understanding the dynamic behaviour of unmyelinated axons and, thus, signal processing and memory.

### Comparison to existing computational models

Compared to existing models this approach offers several distinct advantages. Although HH models of unmyelinated axons (He et al., 2020; Maxion et al., 2023; Tigerholm et al., 2014; Zhang et al., 2008) focus on incorporating biophysical details, our model prioritizes computational efficiency and interpretability. Despite its simplicity it captures essential dynamics, including RCs and PA. It can give insights into underlying molecular processes by correlating axonal dynamics with known biological processes, as discussed above.

One of the most significant advantages of our model is its fast computation time, making it suitable for real‐time applications. The model's computation times range from 2 to 100 ms, depending on the stimulation protocol, whereas the HH model with accurate fibre morphology, such as biologically realistic ion channel subtypes and an axon length of 10 cm with different properties for branch and parent axon, used in a previous study (Maxion et al., 2023), can take up to 20 min for the same protocols.

### Usability

The model fast and effectively predicts spiking times of unmyelinated axons, and its dynamics can be used to interpret underlying molecular mechanisms, making it especially valuable in settings where time is crucial, such as simulations with long computation times in classical HH models or experimental settings.

Simulations of longer axons, such as peripheral axons that can be up to 1 m in length, or complex neural systems are faster and can be implemented through an iterative loop, where each simulation's output serves as the next input. This enables continuous modelling of neuronal activity along extended axonal pathways, which in the case of sensory neurons may reach up to the spinal cord, allowing for parallel simulations that assess synchronization or dyssynchronization crucial for sensory synaptic transmission and processing. This approach is relevant not only for pain but also for myelinated nerve fibres in other sensory systems, potentially aiding in prothesis development. For more complex neural systems involving multiple fibres with varying propagation properties the memory function can be tailored to fit each individual fibre.

This customization necessitates an optimization process similar to what is required for HH models. During this optimization phase the model undergoes numerous evaluations to identify the optimal solution. However due to our model's faster computation times and fewer parameters requiring optimization this process takes only 1–2 min and is therefore significantly quicker than that of traditional HH models, where the same process would take multiple days of computation time.

Once these customized models are established they can be interconnected, using the output from one neuron as the input for the subsequent neuron. This approach enables a comprehensive simulation of interactions within networks of interconnected neurons.

Furthermore the model's ability to represent both short and long ISIs highlights its versatility in addressing diverse experimental conditions, making it a valuable tool for both experimental analysis and hypothesis generation.

Additionally it could assist during experiments by rapidly predicting spike times, both in real time and during postexperiment analysis. In non‐standard stimulation protocols accurately predicting AP latencies can be challenging, complicating the identification of spikes during the experiment. This computational model offers a rapid and efficient method to estimate where APs are likely to occur, aiding in their detection and analysis. In the future these predictions might be incorporated into probabilistic decision‐making layers for automatic spike detection and spike sorting for multifibre recordings.

Further it could be used to simulate stimulation protocols that cannot be practically or ethically performed, e.g. during microneurography in humans, e.g. high‐frequency protocols that cause pain in volunteers.

The model's simplicity also makes it highly adaptable to other axonal types, e.g. sympathetic fibres, which are involved in various pathologies, including vegetative nerve fibres controlling vital functions such as heart rate and blood pressure or axons of the central nervous system. Considering Bostock et al. (2003) it would be feasible to fit our convolution model to various types of units described in their study, such as cold units and sympathetic fibres, more easily and with less computational intensity than classical HH models require. This adaptability allows researchers to explore diverse physiological responses across different unmyelinated fibre types without being hindered by lengthy fitting processes typically associated with conventional models.

In our previous work (Maxion et al., 2023) we used an HH model to assess altered mechanisms in patients with small fibre neuropathy. The novel convolution model can provide mechanistic information through the memory function, which reflects the three phases of axonal excitability that are governed by the history of activity: refractory period, supernormal period and subnormal period (see Fig. 3). After the memory function has been fitted to a patient's spike‐train data, differences in its shape compared to a control group may provide insights into the mechanisms governing theses phases. For example a steeper slope of the refractory period suggests a faster recovery from inactivation of sodium channels. A pronounced supernormal phase could indicate a larger extracellular potassium accumulation and, therefore, differences in potassium channels, and a more pronounced subnormal phase points to more sodium accumulation, which could imply differences in Na^+^/K^+^‐pump activity.

These strengths position the model as a complementary tool to existing frameworks, particularly when rapid computation and flexibility are required.

### Limitations

Despite its strengths the model has several limitations. First defining an accurate memory function remains challenging, as it requires balancing simplicity and fidelity to experimental data. The optimization process, while effective, may be computationally intensive, particularly when integrating more complex memory functions. Additionally the model does not explicitly incorporate information about specific ion channels, limiting its ability to offer mechanistic explanations for observed phenomena onto an indirect approach via dynamics.

In its current state the model would not be easily adjustable to patient data by a technician or nurse without additional support. To facilitate its use in a clinical setting we believe that the development of a user interface is essential. Such an interface would provide the necessary tools and guidance, enabling personnel with typical laboratory training to adapt the model for specific patients.

## Conclusion

This study presents a computational model that effectively captures the dynamics of axonal memory in unmyelinated axons, offering a systematic approach to understanding signal processing along unmyelinated axons. Among the two approaches tested the two‐dimensional memory function, depending on the interstimulus intervals and the PA, emerged as the most accurate. It demonstrated robust performance across a variety of stimulation protocols, including protocols not used in the optimization process, while remaining computationally efficient.

The model's utility lies in its ability to test predictions about axonal memory in a controlled and efficient manner, which is not feasible through experimental methods alone due to the significant time investment required and technical restrictions. By integrating predictions from the model with experimental observations researchers can gain deeper insight into membrane processes shaping AP propagation and their physiological and pathological changes.

One of the key advantages of the model is its computational efficiency, possibly enabling real‐time applications, e.g. predicting AP timing during axonal recordings such as microneurography experiments. This capability is particularly valuable for analysing non‐standard stimulation protocols or unstimulated activity, where latency prediction remains challenging. Consequently, the model could be a valuable addition to spike detection and sorting algorithms.

Moreover its adaptability makes it a promising candidate for future enhancements, in particular adjustments to model different fibre types (e.g. unmyelinated and different myelinated axons) or to model diseased patient‐specific abnormal axonal behaviour.

## Additional information

## Competing interests

J.T has a non‐controlling interest in the company Inventors Way, which supplies research equipment and software. A.M., B.N. and E.K. have no conflicts of interest to declare.

## Author contributions

A.M.: formal analysis, software, visualization, writing – original draft, writing – review and editing. J.T.: supervision, writing – review and editing. B.N.: conceptualization, data curation, supervision, writing – review and editing. E.K.: conceptualization, methodology, supervision, writing – review and editing.

## Funding

B.N.: The work was supported by the German Research Council (DFGNA9706‐2; DFGNA9707‐1; DFGNA9709‐2).

## Generative AI statement

The authors used the generative artificial intelligence (AI) language model RWTHgpt (OpenAI GPT‐OSS 120 b) to proofread the manuscript for grammatical and spelling errors and to perform language editing, as the authors are not native English speakers. Prompts and the resulting outputs were manually inspected by the authors, and only edits that preserved the original scientific meaning were retained. No AI tools were employed for data acquisition, analysis or figure generation. All decisions regarding the final text remain the authors’ responsibility.

## Supporting information




Peer Review History

Supplemental Material

## Data Availability

The code used in this research is available to promote transparency and reproducibility of the results. The complete source code, along with instructions for installation and usage, can be accessed at the repository Digital‐C‐fibre (Maxion et al., 2025). The datasets presented in this article are not readily available because of ethical and privacy restrictions. Requests to access the datasets should be directed to B.N., Namer_B@ukw.de.
