# Supplementary material for: Modelling the memory of unmyelinated axons: Integration of a data‐driven approach with physiological memory concept
Source: J Physiol. 2026 Jul 29;604(17):7125–44. doi: 10.1113/JP290729 (PMC13532846; doi:10.1113/JP290729)
Supplement: Supplementary file 1 [file TJP-604-7125-s001.pdf]

The Journal of Physiology

Modeling the Memory of Unmyelinated Axons: Integration of a Data-Driven Approach with Physiological Memory Concept

Anna Maxion, Jenny Tigerholm, Barbara Namer, and Ekaterina Kutafina
DOI: 10.1113/JP290729

Corresponding author(s): Anna Maxion (anna.maxion@rwth-aachen.de)

The following individual(s) involved in review of this submission have agreed to reveal their identity: Kert Tamm (Referee #1); James Howells (Referee #2)

Review Timeline:

Submission Date:	20-Jan-2026
Editorial Decision:	24-Feb-2026
Revision Received:	05-May-2026
Editorial Decision:	27-May-2026
Revision Received:	05-Jun-2026
Accepted:	12-Jun-2026

Senior Editor: Vaughan Macefield

Reviewing Editor: Theodore Cummins

Transaction Report:

(Note: With the exception of the correction of typographical or spelling errors that could be a source of ambiguity, letters and reports are not edited. Depending on transfer agreements, referee reports obtained elsewhere may or may not be included in this compilation. Referee reports are anonymous unless the Referee chooses to sign their reports.)

Re: JP-RP-2026-290729 "Modeling the Memory of Unmyelinated Axons: Integration of a Data-Driven Approach with Physiological Memory Concept" by Anna Maxion, Jenny Tigerholm, Barbara Namer, and Ekaterina Kutafina

Dear Dr Maxion,

Thank you for submitting your manuscript to The Journal of Physiology. It has been assessed by a Reviewing Editor and by 2 expert referees and we are pleased to tell you that it is potentially acceptable for publication following satisfactory major revision.

Please advise your co-authors of this decision as soon as possible.

The referee reports are copied at the end of this email.

Please address all the points raised and incorporate all requested revisions or explain in your Response to Referees why a change has not been made. We hope you will find the comments helpful and that you will be able to return your revised manuscript within 2 months. If your article is NOT for a Special Issue, you may have 9 months to revise. If you require an extension, please contact journal staff: jp@physoc.org. Please note that this letter does not constitute a guarantee for acceptance of your revised manuscript.

Your revised manuscript should be submitted online using the link in your Author Tasks: Link Not Available. This link is accessible via your account as Corresponding Author; it is not available to your co-authors. If this presents a problem, please contact journal staff (jp@physoc.org). Image files from the previous version are retained on the system. Please ensure you replace or remove any files that are being revised.

If you do not wish to submit a revised version of your manuscript, you must inform our journal staff (jp@physoc.org) or reply to this email to request withdrawal. Please note that a manuscript must be formally withdrawn from the peer review process at one journal before it may be submitted to another journal.

TRANSPARENT PEER REVIEW POLICY: To improve the transparency of its peer review process, The Journal of Physiology publishes online as supporting information the peer review history of all articles accepted for publication. Readers will have access to decision letters, including Editors' comments and referee reports, for each version of the manuscript, as well as any author responses to peer review comments. Referees can decide whether or not they wish to be named on the peer review history document.

ABSTRACT FIGURES: Authors are expected to use The Journal's premium BioRender account to create/redraw their Abstract Figures. Information on how to access this account is here: https://physoc.onlinelibrary.wiley.com/journal/14697793/biorender-access.

This will enable Authors to create and download high-resolution figures. If authors have used the free BioRender service, they can use the instructions provided in the link above to download a high-resolution version suitable for publication.

The link provided should only be used for the purposes of this submission. Authors will be charged for figures created on this account if they are not related to this manuscript submission.

LANGUAGE EDITING AND SUPPORT FOR PUBLICATION: If you would like help with English language editing, or other article preparation support, Wiley Editing Services offers expert help, including English Language Editing, as well as translation, manuscript formatting, and figure formatting at www.wileyauthors.com/eeo/preparation. You can also find resources for Preparing Your Article for general guidance about writing and preparing your manuscript at www.wileyauthors.com/eeo/prepresources.

RIGOUR AND REPRODUCIBILITY: We recommend authors consult The Journal's Rigour and Reproducibility requirements to ensure that their manuscript is suitable for publication: https://physoc.onlinelibrary.wiley.com/pb-assets/hub-assets/physoc/documents/TJP-Rigour-and-Reproducibility-Requirements.pdf

REVISION CHECKLIST:

Check that your Methods section conforms to journal policy: https://jp.msubmit.net/cgi-bin/main.plex?form_type=display_requirements#methods.

Check that data presented conforms to the statistics policy: https://jp.msubmit.net/cgi-bin/main.plex?form_type=display_requirements#statistics.

Upload a full Response to Referees file. To create your 'Response to Referees': copy all the reports, including any comments from the Senior and Reviewing Editors, into a Microsoft Word, or similar, file and respond to each point, using font or background colour to distinguish comments and responses and upload as the required file type.

Please upload two versions of your manuscript text: one with all relevant changes highlighted and one clean version with no changes tracked. The manuscript file should include all tables and figure legends, but each figure/graph should be uploaded as separate, high-resolution files.

You may also upload:

- 'Potential Cover Art' for consideration as the issue's cover image
- Appropriate Supporting Information (Video, audio or data set: see https://jp.msubmit.net/cgi-bin/main.plex?form_type=display_requirements#supp).

We look forward to receiving your revised submission.

If you have any queries, please reply to this email and we will be pleased to advise.

Yours sincerely,

Vaughan Macefield
Senior Editor
The Journal of Physiology

REQUIRED ITEMS

- Author photo and profile. First or joint first authors are asked to provide a short biography (no more than 100 words for one author or 150 words in total for joint first authors) and a portrait photograph. These should be uploaded and clearly labelled together in a Word document with the revised version of the manuscript. See Information for Authors for further details.

- You must start the Methods section with a paragraph headed Ethical Approval. If experiments were conducted on humans, confirmation that informed consent was obtained, preferably in writing, that the studies conformed to the standards set by the latest revision of the Declaration of Helsinki and that the procedures were approved by a properly constituted ethics committee, which should be named, must be included in the article file. If the research study was registered (clause 35 of the Declaration of Helsinki), the registration database should be indicated, otherwise the lack of registration should be noted as an exception (e.g. The study conformed to the standards set by the Declaration of Helsinki, except for registration in a database). For further information see: https://physoc.onlinelibrary.wiley.com/hub/human-experiments.

- Please upload separate high-quality figure files via the submission form.

- Please ensure that any tables are editable and in Word format, and wherever possible, embedded in the article file itself.

- Please ensure that the Article File you upload is a Word file.

- Your paper contains Supporting Information of a type that we no longer publish, including supplementary tables and figures. Any information essential to an understanding of the paper must be included as part of the main manuscript and figures. The only Supporting Information that we publish are video and audio, 3D structures, program codes and large data files. Your revised paper will be returned to you if it does not adhere to our Supporting Information Guidelines.

- Papers must comply with the Statistics Policy: https://jp.msubmit.net/cgi-bin/main.plex?form_type=display_requirements#statistics.

In summary:

- If n {less than or equal to} 30, all data points must be plotted in the figure in a way that reveals their range and distribution. A bar graph with data points overlaid, a box and whisker plot or a violin plot (preferably with data points included) are

acceptable formats.

- If n > 30, then the entire raw dataset must be made available either as supporting information, or hosted on a not-for-profit repository, e.g. FigShare, with access details provided in the manuscript.

- 'n' clearly defined (e.g. x cells from y slices in z animals) in the Methods. Authors should be mindful of pseudoreplication.

- All relevant 'n' values must be clearly stated in the main text, figures and tables.

- The most appropriate summary statistic (e.g. mean or median and standard deviation) must be used. Standard Error of the Mean (SEM) alone is not permitted.

- Exact p values must be stated. Authors must not use 'greater than' or 'less than'. Exact p values must be stated to three significant figures even when 'no statistical significance' is claimed.

- Please include an Abstract Figure file and an Abstract Figure legend. An appropriate figure legend, which should not exceed 150 words in length, should be included in the main manuscript file. The Abstract Figure is a piece of artwork designed to give readers an immediate understanding of the research and should summarise the main conclusions. If possible, the image should be easily 'readable' from left to right or top to bottom. It should show the physiological relevance of the manuscript so readers can assess the importance and content of its findings. Abstract Figures should not merely recapitulate other figures in the manuscript. Please try to keep the diagram as simple as possible and without superfluous information that may distract from the main conclusion(s). Abstract Figures must be provided by authors no later than the revised manuscript stage and should be uploaded as a separate file during online submission labelled as File Type 'Abstract Figure'. Please also ensure that you include the figure legend in the main article file. All Abstract Figures should be created using BioRender. Authors should use The Journal's premium BioRender account to export high-resolution images. Details on how to use and access the premium account are included as part of this email.

- The corresponding author must provide an institutional email address (not a personal address) for their author account. We encourage ALL co-authors to also provide institutional email addresses. If this cannot be provided (as corresponding author), then a stamped letter must be provided from the institution which confirms their role and employment there (please upload this with the revised submission).

EDITOR COMMENTS

Reviewing Editor:
Both reviewers felt that the manuscript was interesting and provided a new method and model for investigating axonal signaling and the dynamic activity of nerve fibers. However, the manuscript could be improved by addressing the comments of the two expert reviewers. Reviewer 2 in particular requests additional insight into the physiological importance of the new model.

The materials and methods section should start with an ethics section with the appropriate information on the human studies listed as per the guidelines:

https://physoc.onlinelibrary.wiley.com/hub/human-experiments

Senior Editor:

Thank you for submitting your manuscript to The Journal of Physiology. I have now received comments form two independent reviewers, both experts in the field, and a reviewing editor. As you will see from their comments, both reviewers see merit in your study but, in comments to me, believe you need to justify how it could realistically be used to gain physiological insight. Nevertheless, I would like to offer you the opportunity to revise your manuscript and provide detailed point-by-point reponses to the reviewers. I look forward to receiving your revised manuscript in due course.

REFEREE COMMENTS

Referee #1:

Review of paper "Modeling the Memory of Unmyelinated Axons: Integration of a Data-Driven Approach with Physiological Memory Concept" submitted to The Journal of Physiology.

In the submitted paper, the authors construct a mathematical model around the concept of process memory using experimental measurements from 95 healthy human patients. The underlying motivation is, in part, computational efficiency, and the authors make a claim in their introduction claiming that their "... highly efficient model enables rapid simulation and analysis that are not feasible with Hodgkin-Huxley models".

This reviewer has reviewed a previous iteration of this paper when it was under consideration for publication in "Journal of Neural Engineering". This version has been improved compared to the previous iteration, and all the comments the reviewer had for the previous iteration have been addressed.

Overall impression of the submitted paper is still positive. The underlying topic appears to be interesting for a wide enough segment of the scientific community, and the authors provide sufficient detail in their paper for a reader who is not intimately familiar with the exact topic under study to roughly understand what and why is done in the submitted paper and what the results are. While in principle the paper is good enough quality for publication, especially after the improvements done to the manuscript compared to the previous iteration, there is only a single remaining minor remark.

In the introduction, the authors make a claim about "... highly efficient model enables rapid simulation and analysis that are not feasible with Hodgkin-Huxley models". - In the reviewer's opinion, that claim is still not properly supported by the following manuscript, even after the improvements which have been made to the section compared to the previous version. This is not a critical issue, just something that caught the eye of the reviewer. In particular, in the reviewer's impression, "not feasible" part of the quoted sentence could be considered perhaps a bit too ambitious, as, in principle, this type of analysis is possible also with a full HH model (even some of the references given in the manuscript), as far as the reviewer understands what has been done in this paper. It would be just significantly more complicated because of a very large number of parameters in the full HH model (as has been pointed out also in this manuscript). The reviewer admits that perhaps he is misunderstanding something here. If the authors aim to emphasise the "rapid simulation and analysis" part of the sentence, not that this type of analysis is not feasible with the HH model, then the reviewer is content to leave this as it is as well. It is not a critical issue, just something that caught the eye.

Referee #2:

This is a nice study which aims to speed up the computation of conduction velocity in unmyelinated axons capturing propagation changes based on previous activity without modelling ion channel behaviour. The comments below are intended to be constructive, and hopefully will help the authors draw more from their study.

1. What I didn't get from the Introduction & Discussion is how this model's advantage of speed could be utilised in practice. Because it doesn't model ion channel behaviour & axonal morphology, it is dependent on capturing changes in conduction velocity by fitting a memory function. That is a valid approach, but it means that to capture a new phenomenon in the same cohort, or to compare across different cohorts a new memory function has to be fitted. This fitting process is clearly computationally intensive, which the authors acknowledge, but may impact the model's utility in practice. It would be nice if the authors could provide a more specific justification of how this model would be used in practice.

a. For example, in the introduction the authors refer to their previous work (Maxion et al 2023) which uses the HH approach to assess altered mechanisms in patients. Could this model provide an advantage in this cohort?

b. Or this there another cohort that the patients could give as an example of the model's utility. Could the authors use their approach for example to model the behaviour of the different types of units in Bostock et al 2003? I am not asking the authors to necessarily do that but rather providing an example for which they may be able to show a potential benefit.

2. In the introduction, the authors state that 'a new model that balances the physiological sophistication of the HH model and the computational efficiency...'. That seems like a totally reasonable aim, and the approach and study provide the mechanisms to achieve that, but the authors don't seem to draw out the physiological insights to back that up. For example, in Figure 3, rather the terms steepness, depth, width and 'moves on x-axis' are fine to describe a mathematical function, but I would argue that you can ascribe some physiological function to them, eg. Steepness is a measure of the recovery from

refractoriness (... related to the dynamics of sodium and fast potassium channels?), depth is the extent of supernormality, (depth and width reflect...), e presumably reflects the time-course of all of the non-linear processes (and is affected by temperature??). So although you do not model ion channel or axonal structure, you should nonetheless be able to make some comparisons between these parameters when fitted in different cohorts or under different conditions.

3. Discussion of the One-Dimensional Memory Function in the Results and Discussion sections could be shortened. It is currently presented in a somewhat 'straw man' manner. It is useful for describing the construction of the 2D model in the Methods section, but the authors state from the outset that they expected the Memory Function to be dependent on prior activity.

4. The Presentation of the model is very clear except for:

a. The use of x & y as variables in the 2D model is unfortunate. Technically, it isn't incorrect, but it is awkward when the 1D model is presented uses t. It would be easier and more intuitive for the reader if the 2D model instead of m(x,y) used t and (say,) PA eg. m(t,PA)

b. In a similar manner, the parameter names are somewhat confusing as presented. For example,

i. c is used to scale the second non-linear term in the 1D model, but is used to scale PA in the linear region of the 2D model.

ii. Similar problems occur with c, f & h.

iii. I suspect that the fitted values for similar parameters (e.g. a, b & d) would differ between the 1D & 2D models. Subscripts may help here (much like you have done with e => e1, e2)

c. It ought to be stated why log(q * y) was used to model the depth of supernormality as a function of PA, is there a reference, or was this from prior observations (eg. linear relationship on a log-scale of pre-existing slowing).

5. Equation 1: (2.2 Specific Memory Function, Line 209)

I think this is a typo, shouldn't the first term be: exp(-d(4t-e) as in a few lines later (Line 216).

ALTHOUGH, I am just double checking that d is not a scaling factor out from of the exponential, ie: -d * exp(4t-e)

Line 239: It might be better to describe parameter g in slightly more physiological terms eg. .... parameter g was introduced to be able to shift the entire memory function up and down.

6. Line 255: replace insulated with isolated

7. Figure 4: Just a suggestion, but Latency increase might be more specific than Latency shift, because it indicates direction.

8. Lines 316 & 321 (2.4 Parameter Optimization). Are the inequality signs the wrong way around on these 2 lines? Isn't the linear part for ISIs >= 0.5s and the non-linear ISIs < 0.5s

9. Line 341. Is the cost function weighted? Eg. giving greater importance for less variable (tighter) experimental data.

10. 3.2.3 and Figure 13 B & D. I would agree that the 2D model captures the character of the dynamics well, but the magnitude does diverge for slowing > ~10%. Can the authors comment on this.

11. Line 560 I am not sure, but the reference (Krishnan and Kiernan 2005) is probably for myelinated axons.

12. Lines 609-611. This is a bit like comparing apples with oranges, and is very dependent on the implementation of the HH model. It would be more valid statement if the authors were able to demonstrate how this model could be used to gain physiological insight.

I was not aware of PlotDigitizer, Thank you!

END OF COMMENTS

EDITOR COMMENTS

Reviewing Editor:
Both reviewers felt that the manuscript was interesting and provided a new method and model for investigating axonal signaling and the dynamic activity of nerve fibers. However, the manuscript could be improved by addressing the comments of the two expert reviewers. Reviewer 2 in particular requests additional insight into the physiological importance of the new model.

The materials and methods section should start with an ethics section with the appropriate information on the human studies listed as per the guidelines:

https://physoc.onlinelibrary.wiley.com/hub/human-experiments

We thank the reviewing editor for their comment. We have moved the ethical statement to the beginning of the methods section in accordance to the guidelines. Additionally, we changed the standard error of the mean to standard deviation in all plots to adhere to journal guidelines.

Senior Editor:

Thank you for submitting your manuscript to The Journal of Physiology. I have now received comments form two independent reviewers, both experts in the field, and a reviewing editor. As you will see from their comments, both reviewers see merit in your study but, in comments to me, believe you need to justify how it could realistically be used to gain physiological insight. Nevertheless, I would like to offer you the opportunity to revise your manuscript and provide detailed point-by-point reponses to the reviewers. I look forward to receiving your revised manuscript in due course.

REFEREE COMMENTS

Referee #1:

Review of paper "Modeling the Memory of Unmyelinated Axons: Integration of a Data-Driven Approach with Physiological Memory Concept" submitted to The Journal of Physiology.

In the submitted paper, the authors construct a mathematical model around the concept of process memory using experimental measurements from 95 healthy human patients. The

underlying motivation is, in part, computational efficiency, and the authors make a claim in their introduction claiming that their "... highly efficient model enables rapid simulation and analysis that are not feasible with Hodgkin-Huxley models".

This reviewer has reviewed a previous iteration of this paper when it was under consideration for publication in "Journal of Neural Engineering". This version has been improved compared to the previous iteration, and all the comments the reviewer had for the previous iteration have been addressed.

Overall impression of the submitted paper is still positive. The underlying topic appears to be interesting for a wide enough segment of the scientific community, and the authors provide sufficient detail in their paper for a reader who is not intimately familiar with the exact topic under study to roughly understand what and why is done in the submitted paper and what the results are. While in principle the paper is good enough quality for publication, especially after the improvements done to the manuscript compared to the previous iteration, there is only a single remaining minor remark.

In the introduction, the authors make a claim about "... highly efficient model enables rapid simulation and analysis that are not feasible with Hodgkin-Huxley models". - In the reviewer's opinion, that claim is still not properly supported by the following manuscript, even after the improvements which have been made to the section compared to the previous version. This is not a critical issue, just something that caught the eye of the reviewer. In particular, in the reviewer's impression, "not feasible" part of the quoted sentence could be considered perhaps a bit too ambitious, as, in principle, this type of analysis is possible also with a full HH model (even some of the references given in the manuscript), as far as the reviewer understands what has been done in this paper. It would be just significantly more complicated because of a very large number of parameters in the full HH model (as has been pointed out also in this manuscript). The reviewer admits that perhaps he is misunderstanding something here. If the authors aim to emphasise the "rapid simulation and analysis" part of the sentence, not that this type of analysis is not feasible with the HH model, then the reviewer is content to leave this as it is as well. It is not a critical issue, just something that caught the eye.

We thank the reviewer for their kind comment. We have revised the sentence in the introduction to clarify that while the simulation itself is indeed feasible in Hodgkin-Huxley models, we intended to say that in our model it is faster. The revised sentence is now stated as follows:

"*With fewer parameters required for fitting diverse datasets, including patient data, our highly efficient model enables faster simulation and analysis of spike train propagation over long distances than Hodgkin-Huxley models, and is therefore suitable of modelling peripheral axons, that can reach axons length up to 1m.*"

Referee #2:

This is a nice study which aims to speed up the computation of conduction velocity in unmyelinated axons capturing propagation changes based on previous activity without modelling ion channel behaviour. The comments below are intended to be constructive, and hopefully will help the authors draw more from their study.

1. What I didn't get from the Introduction & Discussion is how this model's advantage of speed could be utilised in practice. Because it doesn't model ion channel behaviour & axonal morphology, it is dependent on capturing changes in conduction velocity by fitting a memory function. That is a valid approach, but it means that to capture a new phenomenon in the same cohort, or to compare across different cohorts a new memory function has to be fitted. This fitting process is clearly computationally intensive, which the authors acknowledge, but may impact the model's utility in practice. It would be nice if the authors could provide a more specific justification of how this model would be used in practice.
a. For example, in the introduction the authors refer to their previous work (Maxion et al 2023) which uses the HH approach to assess altered mechanisms in patients. Could this model provide an advantage in this cohort?
b. Or this there another cohort that the patients could give as an example of the model's utility. Could the authors use their approach for example to model the behaviour of the different types of units in Bostock et al 2003? I am not asking the authors to necessarily do that but rather providing an example for which they may be able to show a potential benefit.

We are grateful for the reviewer's feedback, which prompts us to elaborate on how the model could be utilized in practice in the Introduction and Discussion. We rewrote the corresponding part of the introduction as follows:

The proposed model is designed to be computationally fast and efficient by excluding detailed ion channel dynamics and other biophysical mechanisms. This makes it capable of simulating spike train propagation in longer axons and complex neural systems and could further make it a valuable tool to predict and sort spikes in real-time during experiments. It simulates how the timing of APs changes while they propagate along the nerve fiber, which makes it suitable for predictive modelling and aiding in the analysis of experimental data. Therefore, the model could enhance our understanding of nociceptive signaling mechanisms and contribute to a broader comprehension of central nervous system functions.

Further, we made several changes to the Discussion, especially in the section "Usability":

1. We clarified that the model is especially useful in time sensitive settings, where fast simulation and prediction is important:

The model fast and effectively predicts spiking times of unmyelinated axons and its dynamics can be used to interpret underlying molecular mechanisms, making it especially valuable in settings where time is crucial, such as simulations with long computation times in classical HH models or experimental settings.

2. We detailed the advantages in the fitting process, i.e. faster computation and fewer parameters, compared to traditional HH model:

   For more complex neural systems involving multiple fibers with varying propagation properties, the memory function can be tailored to fit each individual fiber. This customization necessitates an optimization process, similar to what is required for HH models. During this optimization phase, the model undergoes numerous evaluations to identify the optimal solution. However, due to our model's faster computation times and fewer parameters requiring optimization, this process only takes 1-2 minutes and is therefore significantly quicker compared to that of traditional HH models, where the same process would take multiple days of computation time.

3. We clarified how the model could be fitted to the different fiber types and relate this to the work of Bostock et al. 2003.

   Considering (Bostock et al., 2003b), it would be feasible to fit our convolution model to various types of units described in their study, such as cold units and sympathetic fibers, more easily and with less computational intensity than classical HH models require. This adaptability allows researchers to explore diverse physiological responses across different unmyelinated fiber types without being hindered by lengthy fitting processes typically associated with conventional models.

4. We explained how the model can be used to assess altered mechanisms in patients in comparison to our previous work (Maxion et al. 2023):

   In our previous work (Maxion et al., 2023), we used a HH model to assess altered mechanisms in small fiber neuropathy patients. The novel convolution model can provide mechanistic information through the memory function, which reflects the three phases of axonal excitability that are governed by the history of activity: refractory period, supernormal period and subnormal period (see Figure 3). After the memory function has been fitted to a patient's spike-train data, differences in its shape in comparison to a control group can give insights into the mechanisms governing theses phases. For example, a steeper slope of the refractory period suggests a faster recovery from inactivation of sodium channels, a pronounced supernormal phase could indicate a larger extracellular potassium accumulation and therefore differences in potassium channels, and a more pronounced subnormal

phase points to more accumulated sodium, which could imply differences in Na^{+}/K^{+}-pump activity.

2. In the introduction, the authors state that 'a new model that balances the physiological sophistication of the HH model and the computational efficiency...'. That seems like a totally reasonable aim, and the approach and study provide the mechanisms to achieve that, but the authors don't seem to draw out the physiological insights to back that up. For example, in Figure 3, rather the terms steepness, depth, width and 'moves on x-axis' are fine to describe a mathematical function, but I would argue that you can ascribe some physiological function to them, eg. Steepness is a measure of the recovery from refractoriness (... related to the dynamics of sodium and fast potassium channels?), depth is the extent of supernormality, (depth and width reflect...), e presumably reflects the time-course of all of the non-linear processes (and is affected by temperature??). So although you do not model ion channel or axonal structure, you should nonetheless be able to make some comparisons between these parameters when fitted in different cohorts or under different conditions.

We thank the reviewer for this insightful comment. We agree that we need to make the physiological relevance of the model clearer and made several corresponding changes in the manuscript.

We added an explanation how the model achieves the balance between "physiological sophistication and computational efficiency" in the introduction:

Here, we present a new model that balances the physiological sophistication of the HH model and the computational efficiency of a purely data-driven approach by explicitly incorporating "memory" of unmyelinated axons: the influence of past activity on current conduction latency using a convolution-based approach inspired by geophysical wave propagation models (N. van Dalen et al., 2013). This is achieved by developing a "memory" function that utilizes recovery cycle dynamics as its basis. These dynamics are governed by a refractory period, during which it is impossible or more difficult to generate an action potential (AP), followed by a supernormal phase where the next AP occurs faster, and then a subnormal phase where the subsequent AP is slower. The characteristics of these phases are influenced by previous activity and activity-dependent slowing. Furthermore, these phases can be associated with potential underlying mechanisms: for instance, the refractory period may relate to sodium channel inactivation, while the supernormal phase could be linked to the accumulation of extracellular potassium, and the subnormal phase might correspond to intracellular sodium accumulation. Therefore, this framework allows for drawing mechanistic conclusions from the model.

Further, we rewrote the description of the memory function by adding the physiological dynamics and further edited Figure 3 to incorporate these dynamics:

The function shows three distinct phases that are also observed experimentally: a refractory phase where it is difficult or impossible to generate another action potential, a supernormal

phase, in which the next action potential travels faster than the preceding one, and a subnormal phase, reflecting a slowdown of subsequent spikes (Figure 3)

For shorter ISIs (t<0.5 s), the function is exponential, capturing the rapid and transient effects of recent stimulation. The term $exp\ (-d(4t-e))$ *represents an overall exponential decline, and the term* $c*exp\ (-(4t-e)/f1\ ))$ *defines the supernormal phase. The contained parameters adjust the shape and duration of these phases as follows (Figure 3): The parameter **e** moves the function horizontally, corresponding to a shift of the supernormal phase to different ISIs and reflecting the time course of all non-linear processes. The parameter **d** determines the steepness of the beginning of the function, corresponding to how fast the fiber recovers from the refractory phase. The steepness of this slope can be interpreted as a measure of* Na^{+}*-channel inactivation, where a steeper slope suggests a faster recovery from inactivation. The parameter **f1** determines the width and **c** the depth of the supernormal phase, reflecting the extent of the ISIs with increased conduction velocity. The supernormal phase is physiologically linked to the accumulation of extracellular potassium. A pronounced supernormal phase therefore indicates a larger extracellular potassium accumulation.*

*For longer ISIs (t≥0.5 s), the memory function exhibits a subnormal phase, reflecting a slowdown of subsequent spikes and follows a linear decrease with increasing ISI, which can be adjusted with the parameters **a** and **b**. This describes that the influence of previous activity gradually fades over time, leading to a progressive normalization of conduction velocity and can be associated with intracellular sodium accumulation.*

3. Discussion of the One-Dimensional Memory Function in the Results and Discussion sections could be shortened. It is currently presented in a somewhat 'straw man' manner. It is useful for describing the construction of the 2D model in the Methods section, but the authors state from the outset that they expected the Memory Function to be dependent on prior activity.

We thank the reviewer for their constructive feedback. We agree that the discussion of the one-dimensional memory function can be shortened. We therefore moved the sensitivity analysis of the 1D model to the supplementary materials, since the double pulse protocol already shows that the model can not capture the dynamics depending on different prior activity. Further, we shortened the discussion of the 1D model, highlighting only what the model could and could not reproduce. The in-depth discussion of the physiological relevance of the model was moved to the discussion of the 2D model, where it is more relevant.

4. The Presentation of the model is very clear except for:
a. The use of x & y as variables in the 2D model is unfortunate. Technically, it isn't incorrect, but it is awkward when the 1D model is presented uses t. It would be easier and more intuitive for the reader if the 2D model instead of m(x,y) used t and (say,) PA eg. m(t,PA)
b. In a similar manner, the parameter names are somewhat confusing as presented. For

example,
i. c is used to scale the second non-linear term in the 1D model, but is used to scale PA in the linear region of the 2D model.
ii. Similar problems occur with c, f & h.
iii. I suspect that the fitted values for similar parameters (e.g. a, b & d) would differ between the 1D & 2D models. Subscripts may help here (much like you have done with e => e1, e2)
c. It ought to be stated why log(q * y) was used to model the depth of supernormality as a function of PA, is there a reference, or was this from prior observations (eg. linear relationship on a log-scale of pre-existing slowing).

We thank the reviewer for this comment and improved the model presentation as follows:

1. We changed the naming of the variables in the 2D model to t and PA to make it consistent with the 1D model.
2. We switched the names of the parameters c and h in the 2D model to make the naming consistent with the 1D model.
3. We changed the parameter f to f1 and f2 in the 1D and 2D model respectively to emphasize that they are not identical, but one is the reciprocal of the other.
4. We provided the following explanation why the logarithmic function was used to model the depth of supernormality in relation to PA:

   While the logarithmic dependency was not explicitly stated in (Weidner et al., 2002) it can be inferred from the plots presented in their study. Additionally, we explored several alternative functions, including linear and square root functions. However, the logarithmic function provided the best fit for our data.

5. Equation 1: (2.2 Specific Memory Function, Line 209)
I think this is a typo, shouldn't the first term be: exp(-d(4t-e) as in a few lines later (Line 216). ALTHOUGH, I am just double checking that d is not a scaling factor out from of the exponential, ie: -d * exp(4t-e)
Line 239: It might be better to describe parameter g in slightly more physiological terms eg. .... parameter g was introduced to be able to shift the entire memory function up and down.

We thank the reviewer for catching this typo. It should be exp(-d(4t-e), which is now fixed in the manuscript. Further, we now describe the parameter g in more physiological terms. We have revised the sentence as follows:

Additionally, the parameter g was introduced to allow for vertical adjustments of the entire the memory function.

6. Line 255: replace insulated with isolated

We appreciate the reviewer's suggestion to replace 'uninsulated' with 'unisolated.' However, we would like to clarify that 'uninsulated' is indeed the appropriate term in this context, as it specifically refers to the absence of non-conductive coating over the microelectrodes recording surface.

7. Figure 4: Just a suggestion, but Latency increase might be more specific than Latency shift, because it indicates direction.

We appreciate the reviewer's suggestion regarding the terminology. However, we believe that 'latency shift' is more appropriate in this context, as it indicates that the shift can occur in both directions.

8. Lines 316 & 321 (2.4 Parameter Optimization). Are the inequality signs the wrong way around on these 2 lines? Isn't the linear part for ISIs >= 0.5s and the non-linear ISIs < 0.5s

We thank the reviewer for catching this typo and corrected it in the manuscript.

9. Line 341. Is the cost function weighted? Eg. giving greater importance for less variable (tighter) experimental data.

We thank the reviewer for their insightful question regarding the weighting of the cost function. In our analysis, we did not apply any weights to the cost function. All experimental data were treated equally in terms of their contribution to the overall cost. Additionally, we would like to note that standard deviation data was not available for all datasets, e.g. for the Recovery Cycle and Double Pulse Protocol, which was obtained from the literature. This limited our ability to implement a weighted approach. However, we recognize that weighting could be beneficial in emphasizing less variable experimental data, and we will consider this approach in future work. Thank you for bringing this important point to our attention.

10. 3.2.3 and Figure 13 B & D. I would agree that the 2D model captures the character of the dynamics well, but the magnitude does diverge for slowing > ~10%. Can the authors comment on this.

The concerns raised by the reviewer regarding the divergence in magnitude are valid, which encourages us to acknowledge this behavior in the Results section and refine our discussion on this matter:

Added changes in Results:
The computational model was able to capture the dynamics well, even though the magnitude of the changes differs from the real data, especially for distances of 50 ms.

Added changes in Discussion:
Further, the model could capture the dynamics that were introduced by different amounts of pre-existing activity as seen for the double pulse protocol, where the overall dynamics are captured well even though the magnitude of the changes differs from the real data. In our model this dynamic is governed by the logarithmic increase of the supernormal phase

depending on pre-existing activity. This indicates that a logarithmic function does not fully capture the fiber dynamics and another type of dependency may be needed in future iterations of the model to more accurately represent these variations.

11. Line 560 I am not sure, but the reference (Krishnan and Kiernan 2005) is probably for myelinated axons.

We thank the reviewer for their attention to detail. It is true that Krishnan and Kiernan, 2005 studied myelinated axons. We removed the reference from our manuscript.

12. Lines 609-611. This is a bit like comparing apples with oranges, and is very dependent on the implementation of the HH model. It would be more valid statement if the authors were able to demonstrate how this model could be used to gain physiological insight.

We appreciate the reviewer's observation regarding the comparison between our model and the Hodgkin-Huxley (HH) model. We recognize that differences in implementation can significantly affect outcomes and computation time, making direct comparisons challenging. The specific HH model we are referring to for comparison is our own model from a previous study, as referenced. Therefore, we know the exact computation times for each of the stimulation protocols for this HH model, which is characterized by the use of biological realistic ion channels and axon morphology. Especially, the axon length of 10 cm contributes to the long computation time. Nevertheless, this axon length is needed to simulate the propagation of the action potentials along the nerve fiber. This same propagation can now be simulated with the novel convolution model in a fraction of the time. We find it important to show this comparison to highlight that the reduction of computation time for our novel model is substantial, which has multiple advantages: it is easier and faster to fit the model to different data, for example patient data or different fiber types and the model could be used in real-time applications, such as during a microneurography experiment to quickly sort the action potentials to their respective fibers, which is still a major challenge during these experiments. We improved the corresponding text by making it clear which specific HH model is used for the comparison of computation times. It now reads as follows:

The model's computation times range from 2 to 100 ms, depending on the stimulation protocol, whereas the HH model with accurate fiber morphology, such as biologically realistic ion channel subtypes and an axon length of 10 cm with different properties for branch an parent axon, that we used in a previous study (Maxion et al., 2023) can take up to 20 minutes for the same protocols.

To strengthen our argument, we further improved the discussion on how our model can enhance its utility in gaining physiological insights. The discussion of these aspects has been improved throughout the manuscript as detailed in the response to the reviewers comment 1 and 2.

Dear Dr Maxion,

Re: JP-RP-2026-290729R1 "Modeling the Memory of Unmyelinated Axons: Integration of a Data-Driven Approach with Physiological Memory Concept" by Anna Maxion, Jenny Tigerholm, Barbara Namer, and Ekaterina Kutafina

Thank you for submitting your manuscript to The Journal of Physiology. It has been assessed by a Reviewing Editor and by 2 expert referees and we are pleased to tell you that it is acceptable for publication following satisfactory revision.

The referee reports are copied at the end of this email.

Please address all the points raised and incorporate all requested revisions or explain in your Response to Referees why a change has not been made. We hope you will find the comments helpful and that you will be able to return your revised manuscript within 4 weeks. If you require longer than this, please contact journal staff: jp@physoc.org.

Your revised manuscript should be submitted online using the link in your Author Tasks Link Not Available. This link is accessible via your account as Corresponding Author; it is not available to your co-authors. If this presents a problem, please contact journal staff (jp@physoc.org). Image files from the previous version are retained on the system. Please ensure you replace or remove any files that are being revised.

If you do not wish to submit a revised version of your manuscript, you must inform our journal staff (jp@physoc.org) or reply to this email to request withdrawal. Please note that a manuscript must be formally withdrawn from the peer review process at one journal before it may be submitted to another journal.

TRANSPARENT PEER REVIEW POLICY: To improve the transparency of its peer review process The Journal of Physiology publishes online, as supporting information, the peer review history of all articles accepted for publication. Readers will have access to decision letters, including Editors' comments and referee reports, for each version of the manuscript, as well as any author responses to peer review comments. Referees can decide whether or not they wish to be named on the peer review history document.

ABSTRACT FIGURES: Authors are expected to use The Journal's premium BioRender account to create/redraw their Abstract Figures. Information on how to access this account is here: https://physoc.onlinelibrary.wiley.com/journal/14697793/biorender-access.

This will enable Authors to create and download high-resolution figures. If authors have used the free BioRender service, they can use the instructions provided in the link above to download a high-resolution version suitable for publication.

The link provided should only be used for the purposes of this submission. Authors will be charged for figures created on this account if they are not related to this manuscript submission.

LANGUAGE EDITING AND SUPPORT FOR PUBLICATION: If you would like help with English language editing, or other article preparation support, Wiley Editing Services offers expert help, including English Language Editing, as well as translation, manuscript formatting, and figure formatting at www.wileyauthors.com/eeo/preparation. You can also find resources for Preparing Your Article for general guidance about writing and preparing your manuscript at www.wileyauthors.com/eeo/prepresources.

RIGOUR AND REPRODUCIBILITY: We recommend authors consult The Journal's Rigour and Reproducibility requirements to ensure that their manuscript is suitable for publication: https://physoc.onlinelibrary.wiley.com/pb-assets/hub-assets/physoc/documents/TJP-Rigour-and-Reproducibility-Requirements.pdf

REVISION CHECKLIST:

Check that your Methods section conforms to journal policy: https://jp.msubmit.net/cgi-bin/main.plex?form_type=display_requirements#methods

Check that data presented conforms to the statistics policy: https://jp.msubmit.net/cgi-bin/main.plex?form_type=display_requirements#statistics

Upload a full Response to Referees file. To create your 'Response to Referees' copy all the reports, including any comments from the Senior and Reviewing Editors, into a Microsoft Word, or similar, file and respond to each point, using font or background colour to distinguish comments and responses and upload as the required file type.

Please upload two versions of your manuscript text: one with all relevant changes highlighted and one clean version with no changes tracked. The manuscript file should include all tables and figure legends, but each figure/graph should be uploaded as separate, high-resolution files. The journal is now integrated with Wiley's Image Checking service. For further details, see: https://www.wiley.com/en-us/network/publishing/research-publishing/trending-stories/upholding-image-integrity-wileys-image-screening-service

You may also upload:

- 'Potential Cover Art' for consideration as the issue's cover image
- Appropriate Supporting Information (Video, audio or data set: see https://jp.msubmit.net/cgi-bin/main.plex?form_type=display_requirements#supp).

We look forward to receiving your revised submission.

If you have any queries, please reply to this email and we will be pleased to advise.

Yours sincerely,

Vaughan Macefield
Senior Editor
The Journal of Physiology

REQUIRED ITEMS

- Authors are required to fully disclose the use of any Generative AI (GAI) tools used in the preparation of their manuscript. This disclosure should also be included in the 'Methods' section (or a suitable location for article types not requiring Methods), and include the name(s), model and version of AI technology used, and exact details on the purpose for, and methods of, its use.

If no AI tool has been used, this should also be specifically stated within the manuscript.

EDITOR COMMENTS

Reviewing Editor:

The authors have improved the manuscript and added details on the physiological usefulness of the novel model of unmyelinated axonal conduction.

As one reviewer mentioned, there is still a typo that needs to be corrected on line 290 "Electrical pulses given by an insulated constant current stimulator (Digitimer DS7" - the stimulator is considered isolated. It is likely also insulated, but if correct please replace insulated with isolated as the reviewer suggested.

Senior Editor:

Thank you for submitting your revised manuscript to The Journal of Physiology. As you will see, both reviewers are satisfied with your amendments but there is minor typographical error you will need to correct. I look forward to receiving your revision shortly.

REFEREE COMMENTS

Referee #1:

The changes made to the manuscript are acceptable in this reviewer's opinion. In my opinion, this paper is of good enough quality for publication. There is a potential practical application; the study is supported by sufficient data, and the conclusions drawn appear to be reasonable.

Referee #2:

The authors have addressed all of my concerns.

The only issue is the rebuttal point regarding replacing insulated with isolated.

I agree with the authors, Line 252 refers to an uninsulated microelectrode.... - which sounds right to me.

I was referring to the next sentence in that paragraph. Line 255 from my copy of the previous submission referred to:

"Electrical pulses given by an insulated constant current stimulator (Digitimer DS7, ...", which I still think should be isolated.

END OF COMMENTS

REQUIRED ITEMS

- Authors are required to https://jp.msubmit.net/cgi-bin/main.plex?form_type=display_requirements#aigcfully disclose the use of any Generative AI (GAI) tools used in the preparation of their manuscript. This disclosure should also be included in the 'Methods' section (or a suitable location for article types not requiring Methods), and include the name(s), model and version of AI technology used, and exact details on the purpose for, and methods of, its use.

If no AI tool has been used, this should also be specifically stated within the manuscript.

We have added a paragraph in the Methods section that clarifies the usage of AI, as follows:
2.8. Usage of Artificial Intelligence

We used the generative AI language model RWTHgpt (OpenAI GPT-OSS 120 b) to proofread the manuscript for grammatical and spelling errors and to perform language editing, as the authors are not native English speakers. Prompts and the resulting outputs were manually inspected by the authors, and only edits that preserved the original scientific meaning were retained. No AI tools were employed for data acquisition, analysis, or figure generation. All decisions regarding the final text remain the authors' responsibility.

EDITOR COMMENTS

Reviewing Editor:

The authors have improved the manuscript and added details on the physiological usefulness of the novel model of unmyelinated axonal conduction.

As one reviewer mentioned, there is still a typo that needs to be corrected on line 290 "Electrical pulses given by an insulated constant current stimulator (Digitimer DS7" - the stimulator is considered isolated. It is likely also insulated, but if correct please replace insulated with isolated as the reviewer suggested.

We have corrected this typo in the current version.

Senior Editor:

Thank you for submitting your revised manuscript to The Journal of Physiology. As you will

see, both reviewers are satisfied with your amendments but there is minor typographical error you will need to correct. I look forward to receiving your revision shortly.

REFEREE COMMENTS

Referee #1:

The changes made to the manuscript are acceptable in this reviewer's opinion. In my opinion, this paper is of good enough quality for publication. There is a potential practical application; the study is supported by sufficient data, and the conclusions drawn appear to be reasonable.

Referee #2:

The authors have addressed all of my concerns.

The only issue is the rebuttal point regarding replacing insulated with isolated.

I agree with the authors, Line 252 refers to an uninsulated microelectrode.... - which sounds right to me.

I was referring to the next sentence in that paragraph. Line 255 from my copy of the previous submission referred to:

"Electrical pulses given by an insulated constant current stimulator (Digitimer DS7, ...", which I still think should be isolated.

We thank the reviewer for this clarification. This is indeed a typo and is now fixed in the current version.

Dear Ms Maxion,

Re: JP-RP-2026-290729R2 "Modeling the Memory of Unmyelinated Axons: Integration of a Data-Driven Approach with Physiological Memory Concept" by Anna Maxion, Jenny Tigerholm, Barbara Namer, and Ekaterina Kutafina

We are pleased to tell you that your paper has been accepted for publication in The Journal of Physiology.

The last Word (or similar) version of the manuscript provided will be used by the Production Editor to prepare your proof. When this is ready you will receive an email containing a link to Wiley's Online Proofing System. The proof should be thoroughly checked and corrected as promptly as possible.

Authors should note that it is too late at this point to offer corrections prior to proofreading. Major corrections at proof stage, such as changes to figures, will be referred to the Editors for approval before they can be incorporated. Only minor changes, such as to style and consistency, should be made at proof stage. Changes that need to be made after proof stage will usually require a formal correction notice.

All queries at proof stage should be sent to: TJP@wiley.com.

Following copyediting and typesetting the accepted version will be published online.

Yours sincerely,

Vaughan Macefield
Senior Editor
The Journal of Physiology

IMPORTANT POINTS TO NOTE FOLLOWING ACCEPTANCE OF YOUR PAPER:

• IMPORTANT NOTICE ABOUT OPEN ACCESS: To assist authors whose funding agencies mandate immediate public access to published research findings, The Journal of Physiology allows authors to pay an Open Access (OA) fee to have their papers made freely available immediately on publication.

The Corresponding Author will receive an email from Wiley with details on how to register or log in to Wiley Authors where you will be able to place an order.

• You can check if your funder or institution has a Wiley Open Access Account here:
https://authors.wiley.com/author-resources/Journal-Authors/open-access/author-compliance-tool.html

• TRANSPARENT PEER REVIEW POLICY: To improve the transparency of its peer review process, The Journal of Physiology publishes online as supporting information the peer review history of all articles accepted for publication. Readers will have access to decision letters, including Editors' comments and referee reports, for each version of the manuscript as well as any author responses to peer review comments. Referees can decide whether or not they wish to be named on the peer review history document.

• You can help your research get the attention it deserves! Check out Wiley's free Promotion Guide for best-practice recommendations for promoting your work at: www.wileyauthors.com/eeo/guide. You can learn more about Wiley Editing Services which offers professional video, design, and writing services to create shareable video abstracts, infographics, conference posters, lay summaries, and research news stories for your research at: www.wileyauthors.com/eeo/promotion.

• If you would like to receive our 'Research Roundup', a monthly newsletter highlighting the cutting-edge research published in The Physiological Society's family of journals (The Journal of Physiology, Experimental Physiology, Physiological Reports, The Journal of Nutritional Physiology and The Journal of Precision Medicine: Health and Disease), please click this link, fill in your name and email address and select 'Research Roundup':
https://www.physoc.org/journals-and-media/membernews

EDITOR COMMENTS

Thank you for addressing this minor issue. I am pleased to report that your manuscript is now acceptable for publication in The Journal of Physiology.
