# Supplementary material for: Modelling the memory of unmyelinated axons: Integration of a data‐driven approach with physiological memory concept
Source: J Physiol. 2026 Jul 29;604(17):7125–44. doi: 10.1113/JP290729 (PMC13532846; doi:10.1113/JP290729)
Supplement: Supplementary file 2 [file TJP-604-7125-s002.pdf]

8. Supplemental Material

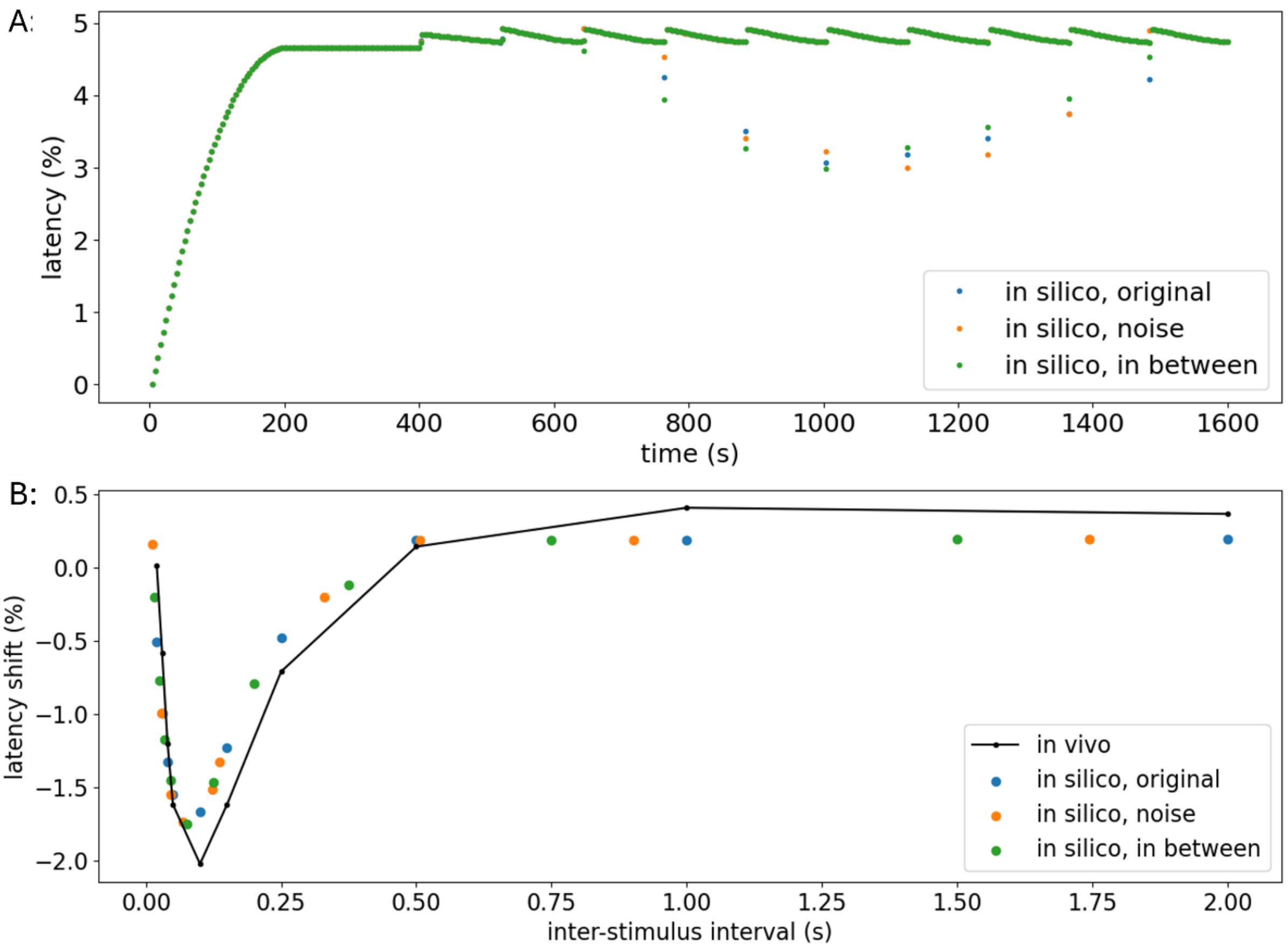

Figure S1: Results of the computational model for the recovery cycle protocol with varying distances between inter-stimulus pulses and a background frequency of ¼ Hz using the one-dimensional memory function. To demonstrate the robustness of the model, the recovery cycle protocol was evaluated under two additional conditions. To assess the model's performance under conditions that mimic experimental variability, random noise was added to the additional pulses in the recovery cycle protocol. The model maintained a high degree of accuracy. The protocol was further tested using ISIs interpolated between the original timings. Again, the model exhibited a strong fit, indicating its ability to accurately predict recovery dynamics across a broader range of conditions. Blue: previously defined inter-stimulus intervals; Orange: Noise is added to the inter-stimulus intervals to get random variations; Green: The distance of the inter-stimulus intervals lies in between the previously defined ones. A: Latency of the action potentials over the time of the stimulation. B: The inter-stimulus interval is shown compared to the relative latency shift induced by the extra pulses. In black the values of the real data from Weidner, 2000, are shown.

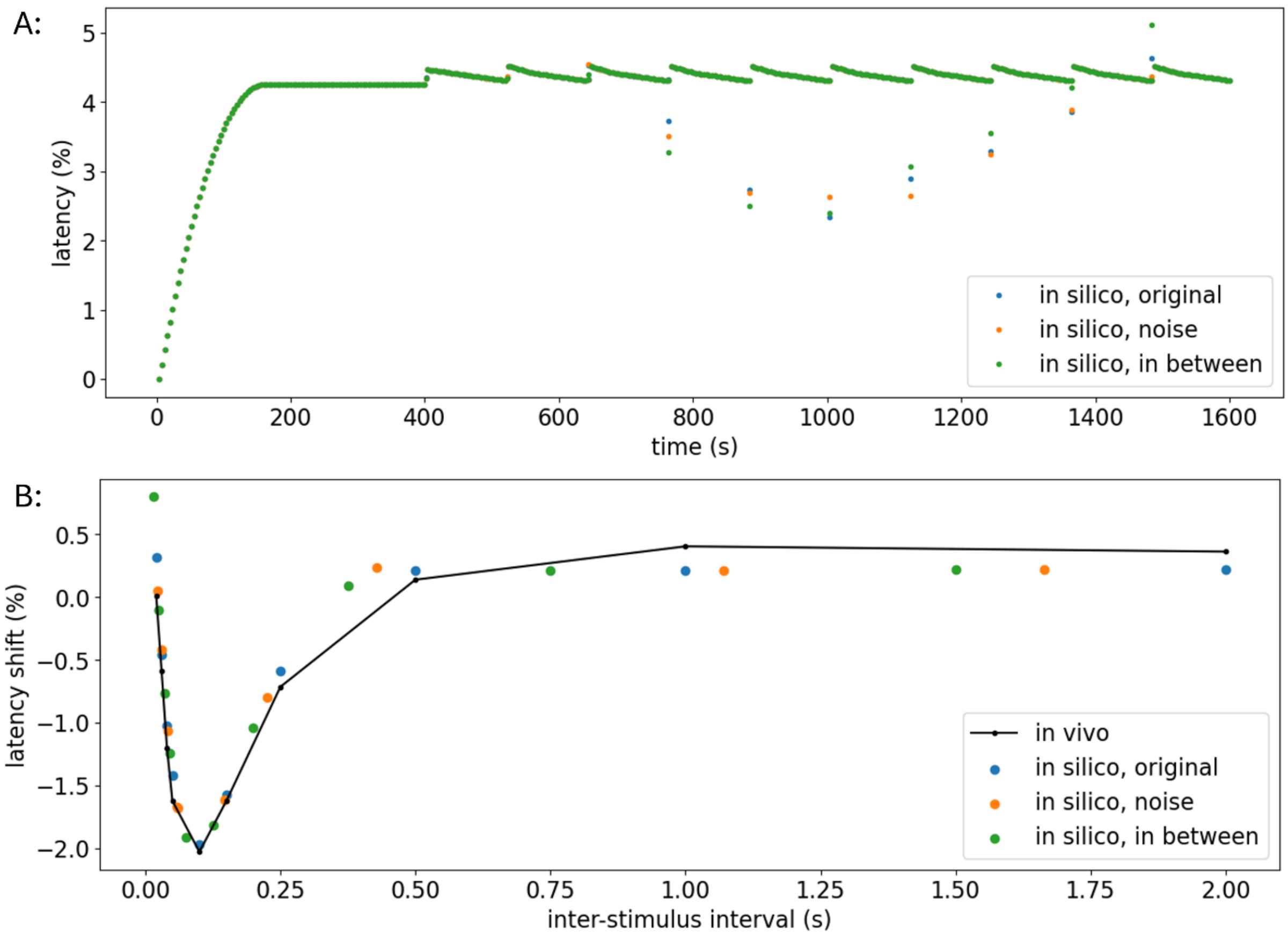

Figure S2: Results of the computational model for the recovery cycle protocol with varying distances between inter-stimulus pulses and a background frequency of ¼ Hz using the two-dimensional memory function. For the sensitivity analysis of the model using the two-dimensional memory function, we tested the recovery cycle protocol with modified ISIs. Both modified protocols could replicate the experimental data well. Blue: previously defined inter-stimulus intervals; Orange: Noise is added to the inter-stimulus intervals to get random variations; Green: The distance of the inter-stimulus intervals lies in between the previously defined ones. A: Latency of the action potentials over the time of the stimulation. B: The inter-stimulus interval is shown compared to the relative latency shift induced by the extra pulses. In black the values of the real data from Weidner, 2000, are shown.

3.1.4. Sensitivity Analysis

Additionally, the recovery cycle protocol was modified by using different frequencies for the background pulses while maintaining consistent ISIs, see Figure S3. This led to varying levels of pre-existing activity state (Figure S3A) but produced the same relative latency shift across all frequencies (Figure S3B). This outcome contrasts with what we would expect in a real

fiber, where the supernormal phase of the recovery cycle depends on the pre-existing activity state and varies with different frequencies.

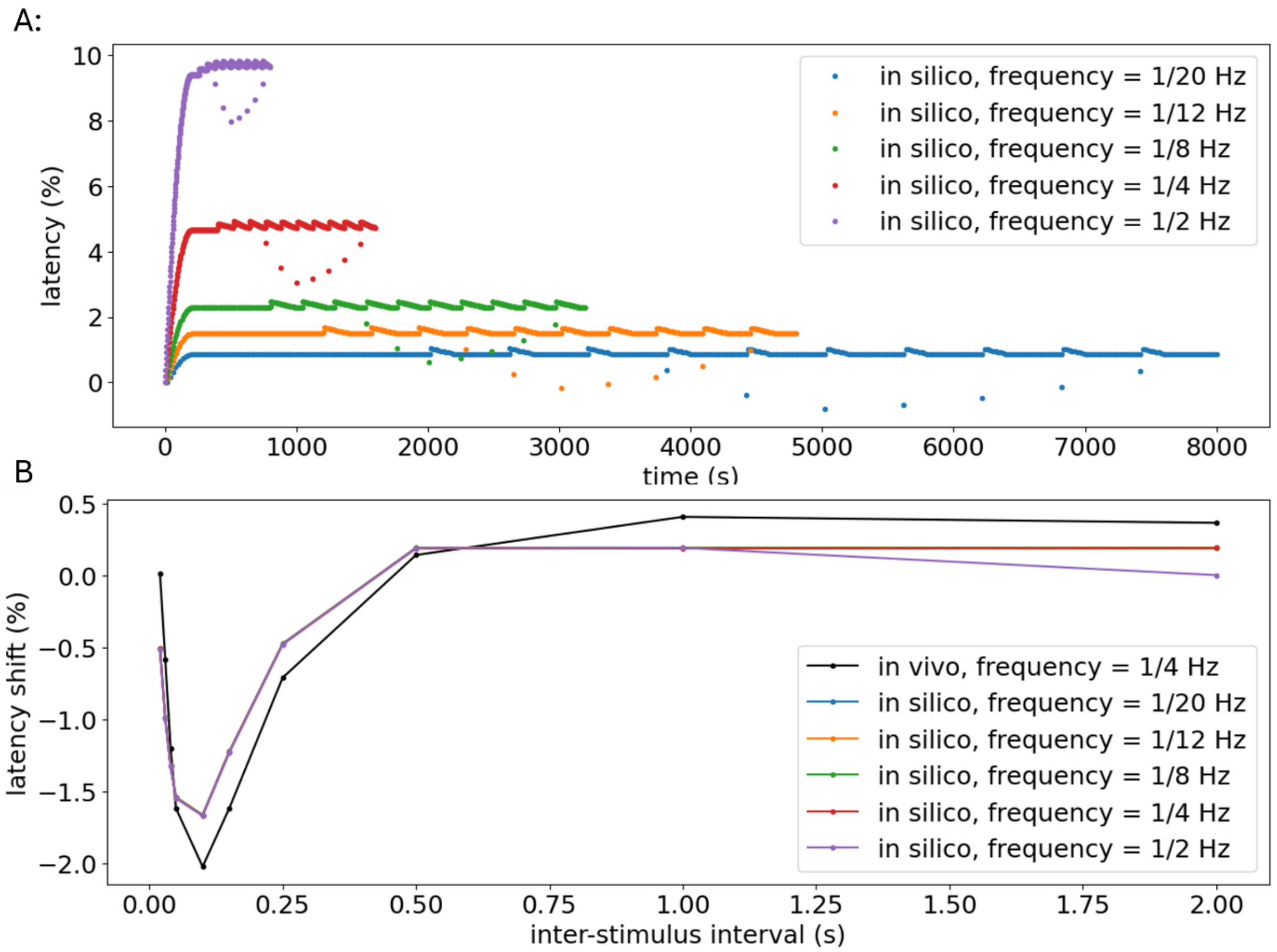

Figure S3: Results of the computational model for the recovery cycle protocol with varying frequencies of the background pulses using the one-dimensional memory function. A: Latency of the action potentials over the time of the stimulation. B: The inter-stimulus interval is shown compared to the relative latency shift induced by the extra pulses. In black the values of the real data from Weidner, 2000, are given for a background frequency of ¼ Hz.

This highlights the limitation of the one-dimensional model. Although the recovery cycle function captures the behaviour of the fiber under various ISIs, it does not account for the effects of pre-existing activity state. When the recovery cycle protocol is performed with a higher frequency of background pulses, the model is not able exhibit the expected more pronounced speeding for small ISIs. Instead, the latency shifts in the recovery cycle protocol remain the same regardless of the background frequency used. This discrepancy suggests that

the current model lacks the ability to represent the full range of dynamic changes that occur with varying pre-existing activity state.
